# A Generalized Linear modeling approach to bootstrapping multi-frame PET image data

**DOI:** 10.1016/j.media.2021.102132

**Published:** 2021-06-12

**Authors:** Finbarr O’Sullivan, Fengyun Gu, Qi Wu, Liam D O’Suilleabhain

**Affiliations:** Department of Statistics, School of Mathematical Sciences, University College Cork, Cork, T12XF62, Ireland

**Keywords:** Bootstrap, Sampling variation, PET, Generalized linear models, Image analysis, Gaussian process, Spectral analysis, Non-parametric methods, Kinetics and residues

## Abstract

PET imaging is an important diagnostic tool for management of patients with cancer and other diseases. Medical decisions based on quantitative PET information could potentially benefit from the availability of tools for evaluation of associated uncertainties. Raw PET data can be viewed as a sample from an inhomogeneous Poisson process so there is the possibility to directly apply bootstrapping to raw projection-domain *list-mode* data. Unfortunately this is computationally impractical, particularly if data reconstruction is iterative or the acquisition protocol is dynamic. We develop a flexible statistical linear model analysis to be used with multi-frame PET image data to create valid bootstrap samples. The technique is illustrated using data from dynamic PET studies with fluoro-deoxyglucose (FDG) and fluoro-thymidine (FLT) in brain and breast cancer patients. As is often the case with dynamic PET studies, data have been archived without raw list-mode information. Using the bootstrapping technique maps of kinetic parameters and associated uncertainties are obtained. The quantitative performance of the approach is assessed by simulation. The proposed image-domain bootstrap is found to substantially match the projection-domain alternative. Analysis of results points to a close relation between relative uncertainty in voxel-level kinetic parameters and local reconstruction error. This is consistent with statistical theory.

## 1. Introduction

Positron emission tomography (PET) is a well-established radiotracer imaging technique, extensively relied on in both secondary and tertiary clinical care settings, as well as in medical research. As the role of quantitative PET in clinical decision making evolves, it is likely that there will be an increasing interest in the availability of practical methods for evaluating uncertainty associated with the results reported for an individual patient. There is already a significant literature on variance assessment for PET. Much of this work has concentrated on the development of analytic approaches based on the linear approximations to the reconstruction process – see, for example, (Alpert et al., 1982; Barrett et al., 1994; Carson et al., 1993; Huesman, 1977; Ibaraki et al., 2014; Maitra and O’Sullivan, 1998; Qi and Leahy, 2000; Tanaka and Murayama, 1982; Wang and Gindi, 1997). The potential of applying Efron’s statistical bootstrap (Efron and Tibshirani, 1994) in this setting was described by (Haynor and Woods, 1989). There have been a number of subsequent contributions - see, for example, (Buvat, 2002; Dahlbom, 2002; Lartizien et al., 2010; Ibaraki et al., 2014; Kucharczak et al., 2018) - that have attempted to implement variations on this approach. The attraction of the non-parametric bootstrap is that it does not involve detailed analytic assumptions which may be difficult to justify in a real patient study. So far, bootstrapping methods for PET image data have concentrated on re-sampling in the raw measurement domain. We refer to such *list-mode* or *sinogram* sampling techniques as projection-domain methods. The work here is stimulated by (Huang et al., 2020), who used a combination of physical phantom studies and numerical simulations to develop an image-domain bootstrapping strategy for PET data. The approach is based on a sub-ordinate Gaussian structure, a particular type of Gaussian copula form (Joe, 2014), with the ability to capture the Poisson-like nature of voxel-level measurements as well as relevant spatial and temporal covariances. In the context of standard clinical PET-FDG studies, involving imaging over a relatively short duration time frame between 45 and 60 minutes after tracer injection, (Huang et al., 2020) proposed sub-dividing frame data in order to obtain the near-replicate information needed to estimate unknown parameters in a proposed image-domain simulation model. This technique was illustrated using data from a clinical PET-FDG lung cancer study.

The work here develops a more flexible procedure for image-domain bootstrapping. This new approach is applicable in situations where there may be a complex temporal structure in the measured PET data – a near-constant temporal structure is intrinsic to the method used in (Huang et al., 2020). In addition the latter work relied on a parametric Gamma-model form to represent the marginal distributions of voxel-level data and a parametric spatial auto-regressive (SAR) form to represent covariance patterns. The method here uses the empirical distribution of re-scaled data and a non-parametric approach for analysis of the spatial correlation structure. The purpose of this report is to describe the modeling techniques involved in a novel image-domain bootstrapping method and to numerically demonstrate its performance relative to the standard projection-domain bootstrapping technique. Similar to (Huang et al., 2020) the proposed approach is only applicable to situations where the PET data have a temporal extent – *e.g.* dynamic PET studies. In this setting suitable modeling of the dynamic data enables us to identify an associated set of residuals that can be manipulated to construct a viable model-based image-domain bootstrapping procedure.

The technical framework for the methodology is set out in Section 2 with some illustrative examples presented in Section 3. The examples come from dynamic studies with PET-FDG and PET-FLT in brain and breast-cancer patients. Apart from the distinctive temporal patterns arising in these data, the studies come from different scanners - one using a traditional analytic filtered-backprojection (FBP) reconstruction method and the other an iterative maximum likelihood (ML) approach. Residual diagnostics demonstrate how the generalized linear modeling adapts to varying nature of the data. A kinetic mapping technique is applied to the bootstrap-simulated datasets in order to produce parametric images of metabolism and associated voxel-by-voxel standard errors. Section 4 presents numerical simulation studies, matched to the real data. These studies explore performance of the novel model-based bootstrapping technique relative to the projection-domain list-mode bootstrapping approach. In addition we examine if performance is impacted by the nature of the data reconstruction process used. The results are very promising, demonstrating that the efficient model-based image-domain bootstrapping substantially matches performance of the projection-domain approach, regardless of what data reconstructed scheme is used. The paper concludes with a discussion in Section 5.

## 2. Methods: Basic models and analysis techniques

The input data for the approach is a 4-D dynamic PET dataset represented by an *N* × *T* array, {*z_ij_*; *i* = 1, ., *N*, *j* = 1, …, *T*} . Here *N* represents the number of voxels in the field of view and *T* is the number of time-frames in the PET acquisition. *z_ij_* is the reconstructed PET-measured tracer concentration value at the 3-D voxel co-ordinate, *x_i_*, at a time *t_j_* corresponding to the mid-point of the *j*’th time-frame of scanning. We begin by providing a formal mathematical description of the model, highlighting the various unknowns that must be estimated before it can be used for bootstrapping.

### 2.1. Statistical modeling of the image-Domain data

Let the true mean and variance of the PET measurement *z_ij_* be denoted *μ_ij_* and $\sigma_{ij}^{2}$, respectively. The proposed approximation of measurements has the structure of a general linear model in which the error process, while allowed to be non-Gaussian and non-stationary, is linked to a sub-ordinate Gaussian process. The starting point for the specification is a set of data-dependent basis vectors, denoted *X* = {*μ_k_*, *k* = 1, 2, …, *K*}, that have the ability to approximate local mean values, *μ_ij_*. The method used to identify *X* is described in Section 2.3 below. The statistical model for *z_ij_* is expressed as a sum of systematic and random terms. The basic version is given by

(1) $$z_{ij}=\mu_{ij}+\sigma_{ij}\epsilon_{ij}\;\;;\;\;\epsilon_{ij}=Q(\eta_{ij})\;\;\text{with}\;\;\eta_{ij}∼N(0,1)\;\mu_{ij}=x_{j}^{\prime }\alpha_{i}\;\;;\;\;\sigma_{ij}=\sigma_{i}\phi_{j}\;\;;\;\;\sum_{j=1}^{T}\phi_{j}^{2}=T$$

Here *x_j_* is the *j*’th row of *X* and *α_i_* is a vector of unknown coefficients. Note that the systematic part of *z_ij_*, here *μ_ij_*, is approximated by a linear form. The model is referred to as a *generalized linear model* because errors are allowed to be non-Gaussian and the scale factors, *σ_ij_*, are not assumed constant (Seber, 2015). In (1), *σ_ij_* is a product of spatial (*σ_i_*) and temporal (*ϕ_j_*) factors. The constraint on *ϕ* is required for identifiability. A key part of (1) is that the error term *ϵ_ij_* is related to a sub-ordinate Gaussian variable, *η_ij_*, by a (unknown) *Q*-transform. In statistical parlance, the inverse of *Q* defines the normal-quantile plot for the collection of measurement errors (R et al., 2020). We assume *Q* is strictly monotone and, for identifiability, scaled so that var(*ϵ_ij_*) = 1. Monotonicity of *Q* is a requirement for the distribution of the measurement errors to be well-defined; it does not restrict the flexibility of the model to adapt to arbitrary distributional forms for the error. Strict monotonicity implies that the quantile mapping is invertible. If the model errors have a continuous distribution - a very plausible approximation for PET and many other types of medical imaging data - then *Q* must be strictly monotone. The sub-ordinate Gaussian *η*-process, *η* = {*η_ij_*, *i* = 1, 2, …, *N*, *j* = 1, 2, …, *T*}, is assumed to have independent temporal components, matching the formal Poisson structure of PET (Vardi et al., 1985), and a common stationary spatial auto-correlation, consistent with physical phantom measurements and simulations, see, for example, (Huang et al., 2020). Note that the concept of using a simple sub-ordinate stationary process, like *η* here, to describe dependency in multivariate data is well established. (Joe, 2014) provides a treatment of many instances which have proved useful in applied statistical work.

While the model in (1) can capture non-stationarity (in mean and variance) and rather general correlation patterns, it has limited ability to adapt to the local skewness of PET data. Such skewness is particularly important for iteratively reconstructed PET data. Thus a more elaborate form of the model is needed for our proposed image-domain bootstrapping procedure. (Scheuermann et al., 2013; Mou et al., 2017) used Gamma-distributions to model the local skewness of iteratively reconstructed PET measurements of scanned physical phantoms. In a Gamma-distribution, the ratio of the mean to the standard deviation is the square-root of the shape parameter. This quantity is inversely proportional to the skewness of the distribution. As the shape parameter increases, skewness diminishes and the Gamma-distribution formally converges to a Gaussian form. Using the fit of the basic model in (1) to define ${\tilde{\mu }}_{ij}$, ${\tilde{\sigma }}_{i}$ and ${\tilde{\phi }}_{j}$, we let $\kappa_{ij}=\frac{{\tilde{\mu }}_{ij}}{{\tilde{\sigma }}_{i}{\tilde{\phi }}_{j}}$. The refined statistical modeling approach uses *κ_ij_* as a surrogate variable for representing deviations from the product form of the variance and the assumed distributional structure. This leads to a more elaborate generalized linear model

(2) $$z_{ij}=\mu_{ij}+\sigma_{ij}\epsilon_{ij}\;\;;\;\;\epsilon_{ij}=Q(\eta_{ij}|\kappa_{ij})\;\eta_{ij}∼N(0,1)\;\mu_{ij}=x_{j}^{\prime }\alpha_{i}\;\;;\;\;\sigma_{ij}=\sigma_{i}\phi_{j}\;\;;\;\;\sum_{j=1}^{T}\phi_{j}^{2}=T$$

where *Q*(·∣*κ*) is strictly monotone for each fixed *κ*-value and is assumed to be slowly varying as a function of *κ*. For identifiability, a scaling constraint involving *Q* is also required. For this with *h*^2^(*κ*) = var(*Q*(*η*∣*κ*)), we require $\sum_{ij}h^{2}(\kappa_{ij})=NT$. Obviously, model (2) reduces to model (1), when the transform *Q* does not vary with *κ*. If *Q*(·∣*κ*) corresponded to a scaled Gamma distribution then, based on (Mou et al., 2017), *κ* would increase with dose or sensitivity. And with increasing *κ*, *Q* becomes linear. Indeed model (2) will converge to model (1), with *Q* ≡ 1, as these factors increase. This is in line with the results reported by (Mou et al., 2017). Thus the structure in (2) gives the ability to adapt to distributions that vary from being highly skewed to ones that are substantially Gaussian. In light of this the model gives the ability to accommodate both the skewed nature of iteratively reconstructed (ML) PET data and the Gaussian nature of analytically reconstructed (FBP) PET scans (Scheuermann et al., 2013; Mou et al., 2017). Regardless of *Q*, the variance structure specified in the model ensures that with particular choices for *σ*, *ϕ* and *h*, the specification in (2) accommodates the situation where variance is directly proportional to the mean - similar to what (Huesman, 1977) proposed for PET ROI data. For this, let *h*(*κ*)^2^ be proportional to ∣*κ*∣, $\phi_{j}={\tilde{\phi }}_{j}$ and choose *σ_i_* proportional to ${\tilde{\sigma }}_{i}$. In this situation the model in (2) could also be viewed within the Gaussian copula framework used by (Lennon and Yuan, 2019) to model time-series of count data.

Since the temporal components of the sub-ordinate Gaussian are assumed independent with a common stationary spatial structure, the covariance of *η* can be written in the form of a Kronecker tensor product, Σ_*N*_ ⊗ *I_T_*, where Σ_*N*_ is a *N* × *N* matrix representing the spatial correlation pattern and *I_T_* is an *T*-dimensional identity matrix. Spatial stationarity implies that the covariance can be diagonalized using the Fourier transform (Brockwell and Davis, 1991), *i.e.* $\sum_{N}=F_{N}^{t}\Lambda_{N}F_{N}$ where $F_{N}$ is the matrix mapping an *N*-vector to its 3-D sine and cosine Fourier coefficients. The action of $F_{N}$ is of course computed using the standard 3D fast Fourier transform (FFT). The matrix Λ_*N*_ is diagonal with elements, *λ* = {*λ_i_*, *i* = 1, …, *N*}. *λ*, which will need to be estimated, has elements corresponding to the discretely sampled 3-D spectral density or power spectrum of *η* (Brockwell and Davis, 1991). While *η* has a stationary spatial structure, the measurement error in either (1) or (2) is neither first nor second order stationary in a spatial sense.

It is important to appreciate that even though the model specification in (2) has substantial flexibility it should only be viewed as a device for obtaining reasonable inferences - here the computation of approximate variances of imaging biomarkers. The general context of modelling in science is worth keeping in mind - approximate models, such as (2), will not be correct to arbitrary precision but in a practical environment they often very useful (Box, 1976). Before providing details of how the various unknowns in (2) are specified, we describe bootstrapping procedures for PET based on our model and also based on a non-parametric model-free approach.

### 2.2. Bootstrapping techniques

Bootstrapping is a general statistical technique that may be used to evaluate the sampling distribution of a statistic, *e.g.* a relevant biomarker, that might be of interest. In particular, the bootstrap sample can be used to obtain bias and variance characteristics of a computed summary statistic, and also to formally access associated hypotheses that might be of interest (Efron and Tibshirani, 1994). Here we consider two possibilities for creating bootstrap samples for PET data: a well-established Projection-Domain approach and the novel Image-Domain approach developed here. The Image-Domain method uses Eqs. (1) and (2); the Projection-Domain is based on the raw count data before it has been reconstructed. It is helpful to record the steps involved in bootstrap simulation by these techniques. This is provided below. We also describe a simple recycling scheme that could be important where it is not practical to retain a large number bootstrap replications.

#### 2.2.1. Projection-Domain bootstrap: Fully non-parametric

This approach involves random sampling (with replacement) from the number (*N_e_*) of detected events. If events are binned into count arrays, this is equivalent to drawing a random sample from a multinomial distribution with *N_e_* trials and probabilities proportional to the observed counts in binned array. Each simulated count array is processed to produce a bootstrap reconstruction, z*. Repeating the process *N_B_* times leads to a projection-domain bootstrap sample, $B_{P}=\{z^{b},b=1,2,\ldots ,N_{B}\}$. If raw data are binned count arrays before reconstruction, there would also be the possibility to sample bootstrap counts using an inhomogeneous Poisson process with mean values proportional to the observed array counts. While this ensures that the simulated data is fully Poisson and more variable than the multinomial, because such sampling would not correspond to *list-mode* re-sampling, we do not use it here. Note that as the count-rate increases, there will be little percentage difference between counts produced by either sampling method.

#### 2.2.2. Image-Domain bootstrap: Model-based approach

Let $\hat{X}$, $\hat{\alpha }$, $\hat{\sigma }$, $\hat{\phi }$, $\hat{\kappa }$, $\hat{Q}$ and $\hat{\lambda }$ be the estimated values of the various unknowns in (2). Bootstrap simulated data, $z_{ij}^{*}$, are generated by first creating an array $\xi^{*}=\{\xi_{ij}^{*},i=1,\ldots ,N,j=1,2.,T\}$ with elements corresponding to a random sample of size *NT* from a standard Gaussian distribution with mean zero and unit variance. With $F_{N}$ representing the normalized 3-D mixed sine and cosine transform (computed by the standard 3-D FFT) and ${\hat{\sigma }}_{ij}={\hat{\sigma }}_{i}{\hat{\phi }}_{j}$, the simulated data are

(3) $$z_{ij}^{*}={\hat{x}}_{j}^{\prime }{\hat{\alpha }}_{i}+{\hat{\sigma }}_{ij}\hat{Q}(\eta_{ij}^{*}|{\hat{\kappa }}_{ij})\;\;\text{where}\;\;\eta_{.j}^{*}=F_{N}^{t}({\hat{\lambda }}^{1∕2}\xi_{.j}^{*})$$

where $\xi_{.j}^{*}$ and $\eta_{.j}^{*}$ are the *j*’th columns of the *N* × *T*-dimensional arrays corresponding to ξ* and η*. Repeating the process *N_B_* times gives an image-domain bootstrap sample, $B_{I}=\{z^{b},b=1,2,\ldots ,N_{B}\}$ where *z^b^* is the *b*’th realization of z* in Eq. (3).

#### 2.2.3. Approximate image-Domain bootstrap by recycling

In practical clinical settings, data retention protocols can mean that raw list-mode data are not routinely archived especially for dynamic studies. In such environments it is also unlikely that there would be willingness to retain extensive bootstrap datasets. Hence a simplified alternative to the full image-domain bootstrap may be of interest. The proposal here is to retain a set of fitted model *α*-coefficients for a small number of image-domain bootstrap samples $\left(N_{B}^{*}\ll N_{B}\right)$ and to base further bootstrap inferences on that dataset - $B_{I}^{*}=\{\alpha^{b},b=1,2,\ldots ,N_{B}^{*}\}$. To justify this, it is important that the coefficients retained are sufficient for the proposed inferences and also that the size of $N_{B}^{*}$ is adequate. Under either of the models in (1,2), any target parameter, *θ*, associated with the measurable tracer concentration signal (*μ_i·_* ≈ *x′α_i_*) is readily expressed in terms of the associated model coefficient - *i.e*. *θ_i_* = *f*(*μ_i·_*) = *f*(*x′α_i_*) ≡ *g*(*α_i_*). Recall that a basic property of maximum likelihood is that if ${\hat{\alpha }}_{i}$ is an optimal estimator of *α_i_* then ${\hat{\theta }}_{i}=g({\hat{\alpha }}_{i})$ is optimal for *θ_i_, c.f.* (Rao, 1973). Thus if the errors in (1) are Gaussian and a corresponding weighted least squares procedure is used for estimation of coefficients, the *α*-coefficient data can be expected to be sufficient in a statistical sense.

We propose a novel *recycling* procedure to help enhance the value of the retained limited data $B_{I}^{*}$. In the approach samples are generated by sampling from $B_{I}^{*}$ a set of say ${\tilde{N}}_{B}$ values at each voxel and then, by application of a spatial filtering process, create a recycled bootstrap dataset ${\tilde{B}}_{I}^{*}=\{{\tilde{z}}^{b},b=1,2,\ldots ,{\tilde{N}}_{B}\}$ where ${\tilde{z}}_{ij}^{b}=x_{j}^{\prime }{\tilde{\alpha }}_{i}^{b}$. Importantly, the recycled ${\tilde{B}}_{I}^{*}$ data is only created at the time that inferences are being considered – so there is no need to retain extensive bootstrap samples. This recycling scheme can be motivated by the fact that the sampling distribution of *α*-coefficient estimates is approximately Gaussian. Note that since the error terms in (1) are independent, Gaussian approximation of the natural weighted least squares estimates of coefficients follows by application of a multivariate version of Lineberg’s central limit theorem - see in Theorem 1 (Bardet et al., 2008). The approximation implies a common Gaussian distribution for the deviations $D_{i}=({\hat{\alpha }}_{i}-\alpha_{i})∕\sigma_{i}=[X^{\prime }WX{]}^{-1}X^{\prime }W^{-1∕2}\epsilon_{i}$ where *W* is the diagonal matrix with elements $w_{j}\propto \phi_{j}^{-2}$ for *j* = 1, 2, …, *T*. Under model (1), *D_i_* is mean zero with covariance *var*(*D_i_*) = Σ_*K*_ = [*X′WX*]^−1^. *D_i_* is a spatially invariant transformation of the error process and also of the underlying sub-ordinate Gaussian process *η*. Thus for (1) the *N* × *K* array, *D*, with rows *D_i_*’s, is strictly stationary in a spatial sense. Now if the error in (1) is exactly Gaussian the covariance of *D* has the Kronecker product form Σ_*N*_ ⊗ Σ_*K*_, where Σ_*N*_ is the spatial covariance of *η*. Thus simulated realizations of *D* can be produced by creating a *N* × *K* array whose rows are independent samples from a *K*-dimensional Gaussian with zero mean vector and covariance Σ_*K*_ and then transforming the columns of the array by multiplication by the matrix $F_{N}^{t}{\hat{\Lambda }}_{N}^{1∕2}$ as in (3). So when Gaussian approximation of $\hat{\alpha }$-coefficients is reasonable, bootstrap samples can be produced by

(4) $${\tilde{\alpha }}_{i}^{b}={\hat{\alpha }}_{i}+{\hat{\sigma }}_{i}{\tilde{D}}_{i}^{b}\;;\;\;i=1,2,\ldots ,N\;b=1,2,\ldots ,{\tilde{N}}_{B}$$

where ${\tilde{D}}^{b}$ is obtained by first sampling, independently for each row, from the distribution of $({\hat{\alpha }}_{i}-\alpha_{i})∕\sigma_{i}$ and then filtering, column-by-column, the resulting *N* × *K* dimensional array using $F_{N}^{t}{\hat{\Lambda }}_{N}^{1∕2}$. Recycling follows this process but uses the empirical distribution of the bootstrap dataset $D_{i}^{*}=\{(\alpha_{i}^{b}-{\hat{\alpha }}_{i})∕{\hat{\sigma }}_{i},b=1,2,\ldots ,N_{B}^{*}\}$ for the sampling from the *i*’th row. An obvious modification of this would be to use the full set of $D_{i}^{*}$’s, $D=\{(\alpha_{i}^{b}-{\hat{\alpha }}_{i})∕{\hat{\sigma }}_{i},b=1,2,\ldots ,N_{B}^{*},i=1,2\ldots ,N\}$, for row-wise simulation. This approach would rely more heavily on the accuracy of the Gaussian approximation for its justification.

### 2.3. Specification of unknowns in the image-Domain model

As indicated earlier, the model in (2) has a number of unknowns all of which need to be defined before image-domain bootstrapping is possible. We begin by describing how the basis set, *X*, is specified and after that consider the other elements.

The goal in basis selection is to choose a configuration that is physiologically interpretable and has the ability to approximate the measured time-course data at all voxels in the volume. A clustering scheme is used to identify clusters of time-courses that have self-similar shapes. If a basis can be found to represent the average time-course in such clusters, then it can be expected that a simple scaling of the representation for the average will fit individual time-course data in the cluster. In light of this, the basis selection is optimized so that the set of cluster means are well represented. But because the number of clusters is quite small (typically on the order of 100–200) relative to the number of voxels, evaluation of the objective function for assessment of any candidate basis set based on the reduced cluster-mean data is computationally efficient. A generalized cross-validation criterion is used as an objective function for basis set assessment. Backwards elimination, a well-established type of greedy algorithm in statistical model selection (Friedman et al., 2001), is used for optimization of the basis. In general the initial basis is taken to be the cluster means. However, in the case that the time-course information is well-sampled in time, a physiologically-based modeling process is used so that instead of raw cluster means being used as the initial basis, a set of model-based predictions of the cluster mean time-courses are used. As some clusters may contain a relatively small number of voxels and as a result may be noisy, the modeling step acts as a well-grounded noise suppression scheme. Modeling also enhances physiologic interpretability of the final set of basis elements selected.

Detailed implementation of the above basis selection scheme substantially follows (O’Sullivan, 1993; O’Sullivan et al., 2014). The process is implemented in two steps. The first step applies recursive hierarchical clustering to partition the data into a large set of clusters, {*C_l_*, *l* = 1, 2, … *L*}, with the property that the data in each cluster has a similar shape pattern: *i.e.* if *i* ∈ *C_l_* then for a suitable constant *a_i_*, *z_ij_* ≈ *a_i_μ_jl_* where ${\tilde{\mu }}_{jl}=\frac{1}{|C_{l}|}\sum_{i\in C_{l}}z_{ij}$ for *j* = 1, 2, …, *T* is the mean time-course for the *l*’th cluster. Step 2 takes the collection of mean time-course patterns associated with each of these clusters as an initial set of basis elements $X_{L}=\{{\tilde{\mu }}_{1},\ldots ,{\tilde{\mu }}_{L}\}$ and applies a cross-validation guided backwards elimination procedure to construct a final subset $X_{K}=\{\mu_{1},\ldots ,\mu_{K}\}\equiv \hat{X}$ with the property that voxel-level data can be adequately represented by a non-negative linear combination of the columns of $\hat{X}$. In the case that well-sampled time-course of PET-measured data is acquired and we have access to the time-course of the tracer in the arterial blood, the second step is modified by replacing each of the cluster mean time-courses by a modeled time-course. A non-parametric residue modeling process is used for that. This is reviewed in Section 3.1 below. Modeling helps to ensure that the final set of basis vectors satisfy physiological constraints linked to the basic principles of blood-tissue exchange (Meier and Zierler, 1954).

#### Specification of the Unknowns, apart from X

With *X* fixed, an unconstrained weighted least squares procedure, with a simple fixed temporal weighting scheme, is used for estimation of *α_i_*’s in (1). The simple weighting scheme and associated *ϕ*-values are

(5) $$w_{j}^{0}=e^{-t_{j}\zeta }dt_{j}∕{\bar{\mu }}_{j}^{*}\;\;\phi_{j}^{0}\propto 1∕\sqrt{w_{j}^{0}}\;\;\text{for}\;\;j=1,2,\;.,\;T$$

Here ${\bar{\mu }}_{j}^{*}=\max({\bar{\mu }}_{j},m)$ where ${\bar{\mu }}_{j}$ is average of the reconstructed concentration values over the *j*’th time-frame and *m* is taken to be a fraction (0.1) of the maximum of these ${\bar{\mu }}_{j}$ values. The duration of the scan time-frame is *dt_j_* and *e^−t_j_ζ^*, with *ζ* the decay constant for the radio-tracer isotope, is the standard tracer decay factor. While the estimates of *α_i_* based on these simple weights may not be optimal, under general conditions weighted least squares estimates are unbiased and also consistent, as the scale of the error diminishes (Seber, 2015). In addition the Gauss-Markov theorem tells us that if weighting is inversely proportional to the variance of the measurement error, least squares will have minimum variance among all unbiased estimators (Seber, 2015). In practice, unless there is a substantial discrepancy between optimal weights and simplistic weights, the latter will typically be highly efficient – see (Romano and Wolf, 2017). In the bootstrapping setting, the unbiasedness property of weighted least squares is important as it ensures that the data simulation process is also unbiased. The use of weighted least squares is also helpful in simply justifying the Gaussian approximation underlying the proposed bootstrap recycling procedure.

Residuals from the least squares fit are used to estimate the other unknowns in (2). The motivation for this comes from

(6) $$r_{ij}\equiv z_{ij}-x_{j}^{\prime }{\hat{\alpha }}_{i}\approx z_{ij}-x_{j}^{\prime }\alpha_{i}=\sigma_{ij}\epsilon_{ij}\equiv \sigma_{i}\phi_{j}\epsilon_{ij}\;\mathrm{so}\;r_{ij}\approx \sigma_{i}\phi_{j}h_{ij}e_{ij}\;\text{with}\;h_{ij}^{2}\;=Var(\in_{ij})$$

and *e_ij_* = *ϵ_ij_*/*h_ij_* has mean zero and unit variance. From (6), natural conditional estimates of *ϕ* and *σ* are

(7) $${\hat{\phi }}_{j}^{2}=\frac{1}{N}\sum_{i=1}^{N}w_{ij}(\sigma ,h)r_{ij}^{2}\;\;\text{and}\;\;{\hat{\sigma }}_{i}^{2}=\frac{1}{T}\sum_{j=1}^{T}w_{ij}(\phi ,h)r_{ij}^{2}$$

where $w_{ij}(\sigma ,h)=\sigma_{i}^{-2}h_{ij}^{-2}$ and $w_{ij}(\phi ,h)=\phi_{j}^{-2}h_{ij}^{-2}$. ${\hat{\phi }}_{j}^{2}$ values are scaled so that $\sum_{j}{\hat{\phi }}_{j}^{2}=T$. For any specified *h*, (7) can be iterated, starting with *σ* constant, to obtain converged values $\tilde{\phi }(h)$ and $\tilde{\sigma }(h)$. These are maximum likelihood estimates when *e_ij_* is standard Gaussian. With *h_ij_* = 1, the converged values in (7), denoted $\tilde{\phi }$ and $\tilde{\sigma }$, are used as estimates of *ϕ* and *σ* in model (1). This is important for specification of *κ*. The set of $\hat{\kappa }$-values are defined by ${\hat{\kappa }}_{ij}=\frac{{\hat{z}}_{ij}}{{\tilde{\sigma }}_{i}{\tilde{\phi }}_{j}}$ where ${\hat{z}}_{ij}=x_{j}^{\prime }{\hat{\alpha }}_{i}$.

For estimation of *Q*, we restrict to piecewise constant approximation as a function of the *κ* variable. With *κ*_(0)_ = −∞ and *κ*_(*l*)_ the $\frac{l}{L}\times 100\%$’th percentile of the ${\hat{\kappa }}_{ij}$ values, let *I_l_* be the interval (*κ*_(*l*−1)_, *κ*_(*l*)_], for *l* = 1, 2, …, *L*. Piecewise constant approximation means $Q(\cdot |\hat{\kappa })=\hat{Q}(\cdot |l)$ for $\hat{\kappa }\in I_{l}$. Our experience is that *Q* is a smooth function of $\hat{\kappa }$ so that a modest value for *L* (in the 50 to 100 range) seems to be quite adequate. As *Q* is piecewise constant, its variance is also piecewise constant - $Var(Q(\eta |\hat{\kappa }))=h(\hat{\kappa }{)}^{2}=h_{l}^{2}$ for $\hat{\kappa }\in I_{l}$. This implies *h_ij_* = *h_l_* for ${\hat{\kappa }}_{ij}\in I_{l}$. Similar to (7), a conditional estimate of *h_l_* given *σ* and *ϕ* is

(8) $${\hat{h}}_{l}^{2}=\frac{1}{|I_{l}|}\sum_{{\hat{\kappa }}_{ij}\in I_{l}}w_{ij}(\sigma ,\phi )r_{ij}^{2}\;\;\text{with}\;\;w_{ij}(\sigma ,\phi )=\sigma_{i}^{-2}\phi_{j}^{-2}$$

normalized so that $\sum_{l}{\hat{h}}_{l}^{2}=L$. Combining (7) and (8), provides an iteration for joint estimation of *σ*, *ϕ* and *h*. This defines $\hat{\sigma }$ and $\hat{\phi }$. The converged $\hat{\sigma }$ values are inflated by multiplication by $\frac{T}{T-K}$ where *K* is the number of columns of *X*. This is to ensure there is adjustment for the bias arising from fitting the *α*-coefficients at each voxel. Such adjustments are standard in most parametric model fitting settings - see (Kutner et al., 2013; Seber, 2015) for its use in linear regression.

The converged values of $\hat{\sigma }$ and $\hat{\phi }$ are used to compute scaled residuals ${\hat{\epsilon }}_{ij}=r_{ij}∕{\hat{\sigma }}_{i}{\hat{\phi }}_{j}$. The empirical distribution of these scaled residuals, $E_{l}=\{{\hat{\epsilon }}_{ij}\;;\;{\hat{\kappa }}_{ij}\in I_{l}\}$, is used to evaluate $\hat{Q}(\cdot |l)$. Here we match order statistics of *E_l_* to the corresponding quantiles of a standard normal distribution - $N_{l}=\{{\hat{\eta }}_{ij}\;;\;{\hat{\kappa }}_{ij}\in I_{l}\}$. This is simply the standard normal quantile-quantile plot procedure - *c.f.* (R et al., 2020). The inverse map allows arbitrary quantiles of the standard normal to be mapped to quantiles of *E_l_*.

For estimation of *λ*, the full set of normalized residuals, $\hat{\eta }$, are mapped to the imaging domain and using the 3-D FFT their 3-D periodogram is evaluated for each time-frame. Averaging these periodograms over time-frames, with weights ${\hat{\phi }}_{j}^{-2}$, produces the final estimate of the required power spectrum, $\hat{\lambda }$. Using the Weiner-Khinchine theorem (Brockwell and Davis, 1991), the inverse 3-D FFT of the power spectrum provides the corresponding of the 3D spatial auto-correlation function $\hat{\rho }$. This completes the specification of all unknowns in the image-domain bootstrapping model. Before investigating the reliability of the bootstrapping method, we first present some illustrations of the technique with real data. Model diagnostics are an essential part of any statistical modeling process, particularly if model-based bootstrapping is of interest. This aspect is highlighted in the examples.

## 3. Applications to parametric imaging

We present two dynamic PET imaging studies of cancer patients. One is from a series reported in (Spence et al., 1998) and involves brain tumor scanning with ^18^F-labeled Fluorodeoxyglucose (FDG); the second comes from a more recent breast cancer imaging trial with ^18^F-labeled Fluorothymidine (FLT) (Kostakoglu et al., 2015). The studies are chosen in part because they represent data with different imaging challenges. The brain FDG study is from an early generation PET scanner using direct FBP reconstruction. The scanner in the FLT study is more recent and uses an ML reconstruction technique. Raw projection data are not available for either study. Thus only image-domain bootstrapping is practical. In each case the bootstrap generated dataset, $B_{I}$, is processed to map metabolic parameters and their associated standard errors. Before presenting the examples, we provide some background on the approach used for mapping voxel-level kinetic parameters. This material draws on (O’Sullivan, 1993; O’Sullivan et al., 2014).

### 3.1. Non-Parametric residue mapping (NPRM) of kinetics

The basic principle of most tracer imaging studies with PET is that the tracer’s interaction with the local tissue is linear and time-invariant. While there are situations where this assumption is not valid, it is very reasonable for FDG and FLT. The arterial supply is the primary system for transport of tracer to tissue, so linearity and time-invariance implies that the true tissue concentration, *C*(*x, t*), can be described by a convolution between the local arterial blood concentration supplied by the output of the left-ventricle (LV) of the heart, *C_p_*, and the corresponding impulse-response, or so-called tissue residue function *R*. Thus

(9) $$C(x,t)=\int_{0}^{t}R(x,t-s)C_{p}(x,s)ds$$

Typically arterial dispersion effects are below the resolution of the PET scanner and the arterial concentration can be well-described by a suitable shift of the LV signal – *C_p_*(*x, s*) = *C_p_* (*s* − Δ_*x*_) where *C_p_*(*t*) is the LV blood concentration and Δ_*x*_ is a suitable delay. From basic principles of blood-tissue exchange (Meier and Zierler, 1954), the residue is a monotone-decreasing non-negative function - a life-table for the travel-times of tracer atoms introduced to tissue at time zero (O’Sullivan et al., 2009). While most tissue in a typical volume of interest behaves in the above manner, some exceptions may need to be kept in mind. For example, there may be areas between the (venous) injection site and the corresponding pathway to the LV where arterial pattern is irrelevant. Similarly, if the bladder is in the field of view, its time-course will not follow the pattern in (9). It is appropriate to describe it in terms of the outflow for a whole-body blood-tissue exchange process (Meier and Zierler, 1954).

There is a substantial literature on modeling techniques for PET time-course data. Most of this focuses on the analysis of region of interest data, see, for example, (Huang et al., 1986; Vicini and Bassingthwaighte, 2014; Mankoff et al., 2006). But there are also a number of techniques for voxel-level analysis. Spectral techniques use a positive linear combination of exponentials to approximate the voxel-level residue (Cunningham and Jones, 1993; Veronese et al., 2016; Wang et al., 2020). The NPRM approach models voxel-level time-course data by a positive linear combination of basis vectors, {*μ_k_*, *k* = 1, …, *K*} – referred to a sub-TACs (TAC stands for time-activity curve). This is a version of the form used in equations (1,2) in which the *α*-coefficients are constrained to be non-negative. In the NPRM setting, the backwards elimination scheme in Section 2.3 is modified by replacing the initial cluster-mean vectors in *X_L_* by elements based on non-parametric residue modeling of the cluster mean data – *i.e.* ${\tilde{\mu }}_{l}$ is replaced by the approximation $\mu_{l}(t)=\int_{0}^{t}R_{l}(t-s)C_{p}(s-\Delta_{l})ds$. In addition, the basis in NPRM is required to always include components corresponding to the time-courses for the arterial input function (AIF) and its cumulative integral. The latter is referred to as the Patlak basis element because an analysis that only used that term would be substantially equivalent to the Patlak approach to evaluation of tracer flux (Patlak and Blasberg, 1985).

As the basis elements are represented in terms of residue functions, $\mu_{k}(t)=\int_{0}^{t}R_{k}(t-s)C_{p}(s-\Delta_{k})ds$, the linear model representation of the PET data leads to the approximation of the voxel-level residue as a linear combination of the basis residues {*R_k_*, *k* = 1, …, *K*}, *i.e.*

(10) $$z_{ij}\approx \sum_{k=1}^{K}\alpha_{ik}\mu_{k}(t_{j})\;\;\to \;\;R(x_{i},t)\approx \sum_{k}\alpha_{ik}R_{k}(t)$$

Here a very short duration (less than 5 seconds) spiked residue is used to represent the AIF, a constant residue is used for the Patlak term.

In the NPRM approach, decomposition of the voxel-level residue is used to create summaries variables for mapping tracer kinetics. Suppose *T_E_* is the study duration and let *T_B_* for 0 < *T_B_* < *T_E_* represent a realistic upper-bound for large-vessel travel-time – for human PET studies with FLT and FDG, a value *T_B_* of around 5 to 10 seconds is reasonable physiologically. The residue over the observed time-frame of the study, [0, *T_E_*], is decomposed in terms of vascular (*R_B_*), in-distribution (*R_D_*) and extraction (*R_E_*) components as

(11) $$R(t,x)=R_{B}(t,x)+R_{D}(t,x)+R_{E}(t,x)$$

where

RE(t,x)=R(TE,x)≡Ki(x)RB(t,x)={R(t,x)−R(TB,x),0≤t≤TB0,elsewhere}RD(t,x)=R(t,x)−RB(t,x)−RE(t,x)

The apparent rate of extraction of tracer atoms by the tissue is measured by *K_i_*(*x*). This is a measure of flux (see further discussion below). Given the residue decomposition, the vascular (large vessel) blood volume, *V_B_*(*x*), as well as the in-distribution flow, *K_D_*(*x*), and volume, *V_D_*(*x*), are recovered from *R_B_* and *R_D_* as

(12) $$V_{B}(x)=\int_{0}^{T_{B}}R_{B}(t,x)dt\;K_{D}(x)=R_{D}(0)\;\;;\;\;V_{D}(x)=\int_{0}^{T_{E}}R_{D}(t,x)dt$$

By the central volume theorem (Meier and Zierler, 1954), the mean transit time (MTT) is defined as the ratio volume to flow – *V_D_*(*x*)/*K_D_*(*x*). However this does not take account of variation in the time of arrival of the tracer to the local tissue. To address this we use a flow-weighted average of mean transit-times associated individual sub-TACs – *i.e.* MTT(*x_i_*) = Σ_*k*_*w_ik_*MTT_*k*_ where MTT_*k*_ = Δ_*k*_ + *V_Dk_*/*K_Dk_* with *K_Dk_* and *V_Dk_* obtained from *k*’th component residue (*R_k_*). Here the weights, which are normalized to sum to unity, are proportional to the flow contributions from the different component tissues represented at the *i*’th voxel, *i.e w_ik_* ∝ *α_ik_K_Dk_* for *k* = 1, 2, … *K*. Thus MTT is written as

(13) $$\text{MTT}(x_{i})=\Delta_{w}(x_{i})+V_{D}(x_{i})∕K_{D}(x_{i})\;\;=\Delta_{w}(x_{i})+\frac{\sum_{k=1}^{K}\alpha_{ik}V_{Dk}}{\sum_{k=1}^{K}\alpha_{ik}K_{Dk}}$$

Δ_*w*_(*x_i_*) = Σ_*k*_*w_ik_*Δ_*k*_. In the case that the individual delays are all the same, Δ_*w*_(*x_i_*) will constant across voxels.

While a non-parametric approach is used for specification of sub-TAC residues, it is useful to record what the above summary parameters correspond to in the 2-compartmental model of Huang and Sokoloff, see (Huang et al., 1986). This model is very widely used in PET data analysis. In this model there are four kinetic constants - (*k*_1_, *k*_2_, *k*_3_, *k*_4_). The impulse response function (*a.k.a* tissue residue) for the model is a mixture of exponentials

(14) $$I_{C}(t)=k_{1}(1-\pi )e^{-t\lambda_{1}}+k_{1}\pi e^{-t\lambda_{2}}$$

where $\lambda_{1(2)}=\frac{1}{2}(k_{2}+k_{3}+k_{4}\pm \sqrt{\left(k_{2}+k_{3}+k_{4}{)}^{2}-4k_{2}k_{4}\right)}$ and $\pi =\frac{k_{3}+k_{4}-\lambda_{2}}{\lambda_{1}-\lambda_{2}}$. When the model is applied to PET time-course data, there is typically an adjustment for the fractional blood volume (*f_b_*), this gives rise to a model representation of the tissue time-course (*C_T_*) as

(15) $$C_{T}(t)\approx f_{b}C_{p}(t)+(1-f_{b})\int_{0}^{t}I_{C}(t-s)C_{p}(s)ds$$

Suppose we introduce a simple linear residue *R_o_* defined over the interval [0, *T_B_*] by

Ro(t)={2fbTB(TB−tTB),0≤t≤TB0,elsewhere}

Note $\int_{0}^{T_{B}}R_{o}(t)dt=f_{b}$. As *T_B_* → 0, *R_o_* becomes very spiked at 0. For small *T_B_* and the convolution of *R_o_* with the AIF is approximately *f_b_C_p_*(*t*). As a result, for sufficiently small *T_B_*, the 2-compartmental model can be given a residue representation

(16) $$C_{T}(t)\approx f_{b}C_{p}(t)+(1-f_{b})\int_{0}^{t}I_{C}(t-s)C_{p}(s)ds\;\;=\int_{0}^{T}R_{C}(t-s)C_{p}(s)ds$$

with *R_C_*(*t*) = *R_o_*(*t*) + (1 − *f_b_*)*I_C_*(*t*). Decomposing *R_C_* as described in Eq. (11) and evaluating the residue summary measures gives

(17) $$K_{i}=K_{1}(1-\pi )e^{-\lambda_{1}T_{E}}+K_{1}\pi +O(\lambda_{2})\;V_{B}=f_{b}+O(T_{B})\;K_{D}=K_{1}-K_{i}+O(T_{B})\;V_{D}=\frac{K_{1}(1-\pi )(1-e^{-\lambda_{1}T_{E}})}{\lambda_{1}}+O(\lambda_{2})+O(T_{B})$$

where *K*_1_ = (1 − *f_b_*)*k*_1_ and *O*(*x*) indicates that corresponding terms vanish as *x* → 0. If *k*_4_ = 0 then *λ*_2_ = 0. Here as *T_B_* → 0 with *T_E_* large (so *e*^−*λ*_1_*T_E_*^ is negligible) the residue summaries for the 2-C model become

(18) $$K_{i}=K_{1}\pi =\frac{K_{1}k_{3}}{k_{2}+k_{3}}\;V_{B}=f_{b}\;K_{D}=K_{1}(1-\pi )=\frac{K_{1}k_{2}}{k_{2}+k_{3}}\;V_{D}=\frac{K_{1}}{k_{2}+k_{3}}(1-\pi )=\frac{K_{1}k_{2}}{(k_{2}+k_{3}{)}^{2}}$$

When both *k*_3_ and *k*_4_ are zero, the 2-compartment model reduces to the 1-compartment Kety-Schmidt model (Kety and Schmidt, 1945). Here the parameters become: *K_i_* = 0 (no retention), *V_B_* = *f_b_*, *K_D_* = *K*_1_ and $V_{D}=\frac{K_{1}}{k_{2}}$. The latter two quantities are the familiar flow and distribution volume terms associated with the Kety-Schmidt approach to the quantitation of PET studies with ^15^O-labeled water.

In the two applications, the basis functions used for generation of bootstrap data are the same as those used for NPRM kinetic analysis of the original image data (*z*). Individual bootstrap realizations $\left(z^{b}\in B_{I}\right)$ are processed in the same way as the original data using the NPRM procedure. If an alternative kinetic or other analysis method was of interest, it would be applied to the original data and to the realizations in $B_{I}$. In this way the bootstrapping technique could also be used to generate assessments of uncertainties for alternative approaches to mapping kinetics, *e.g.* such as those reviewed in (Wang et al., 2020).

### 3.2. FDG Brain tumor study

PET studies with FDG play a major role in the diagnosis and management of many cancers (Barrio et al., 2020). (Spence et al., 1998) reported on a series of NIH-supported studies, conducted at the University of Washington (Seattle), evaluating the ability to measure the metabolic rate of glucose consumption in glioma patients, post-surgery. We use data from one of these cases. Details of the study protocol, which also included direct arterial blood sampling, are provided in (Spence et al., 1998). Briefly, imaging was conducted a 35-plane scanner using a 2-D plane-by-plane acquisition process and a direct (FBP) reconstruction methodology. The 4-D PET data is an array with *N* = 128 × 128 × 35 voxels (2.25 × 2.25 × 4.25*mm*^3^) and *T* = 31 time-frames extending over a 90 minute period. The time-frame sequence is: 1(1 min) preinjection, 4(20 sec), 4(40 sec), 4(1 min), 4(3 min) and 8(5 min). The bootstrapping technique is used to evaluate sampling variation in computed metabolic images.

**Fig. 1.**
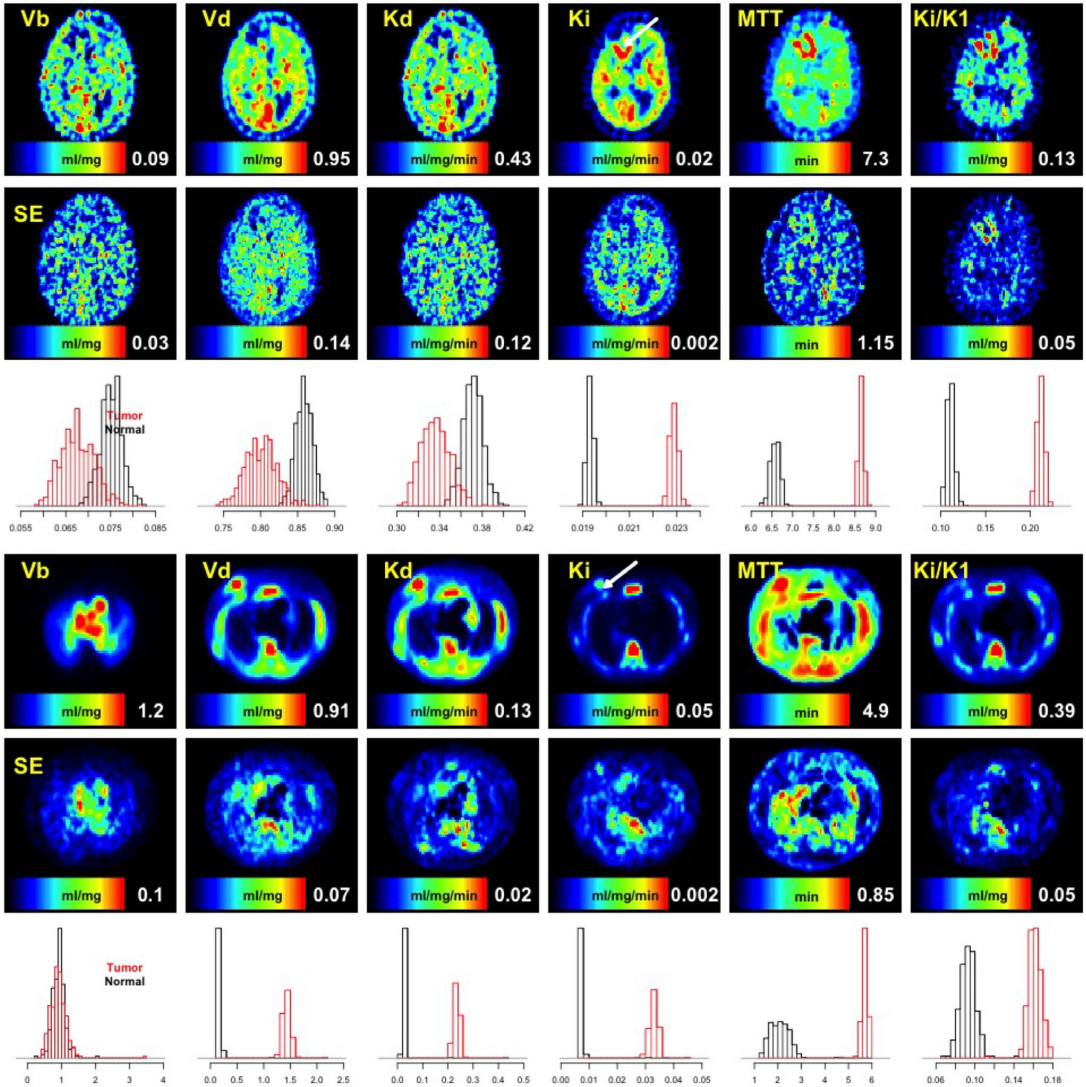
Metabolic Images with image-domain bootstrap assessment of standard errors: Rows 1–3 show the Brain tumor FDG data; 4–6 for the Breast tumor FLT data. The mapped parameters are shown on rows 1&4 – the location of tumor volume of interest (VOI) on the slice is indicated with an arrow. Columns correspond to different metabolic parameters (labeled in yellow) with color bars indicating units - see Section 3.1 for definitions. Computed standard errors (SE) are on rows 2&5. Rows 3&6 show histograms of the bootstrap sampling distributions of the95th percentile of the metabolic parameter in the normal [black] and tumor [red] VOIs.

**Fig. 2.**
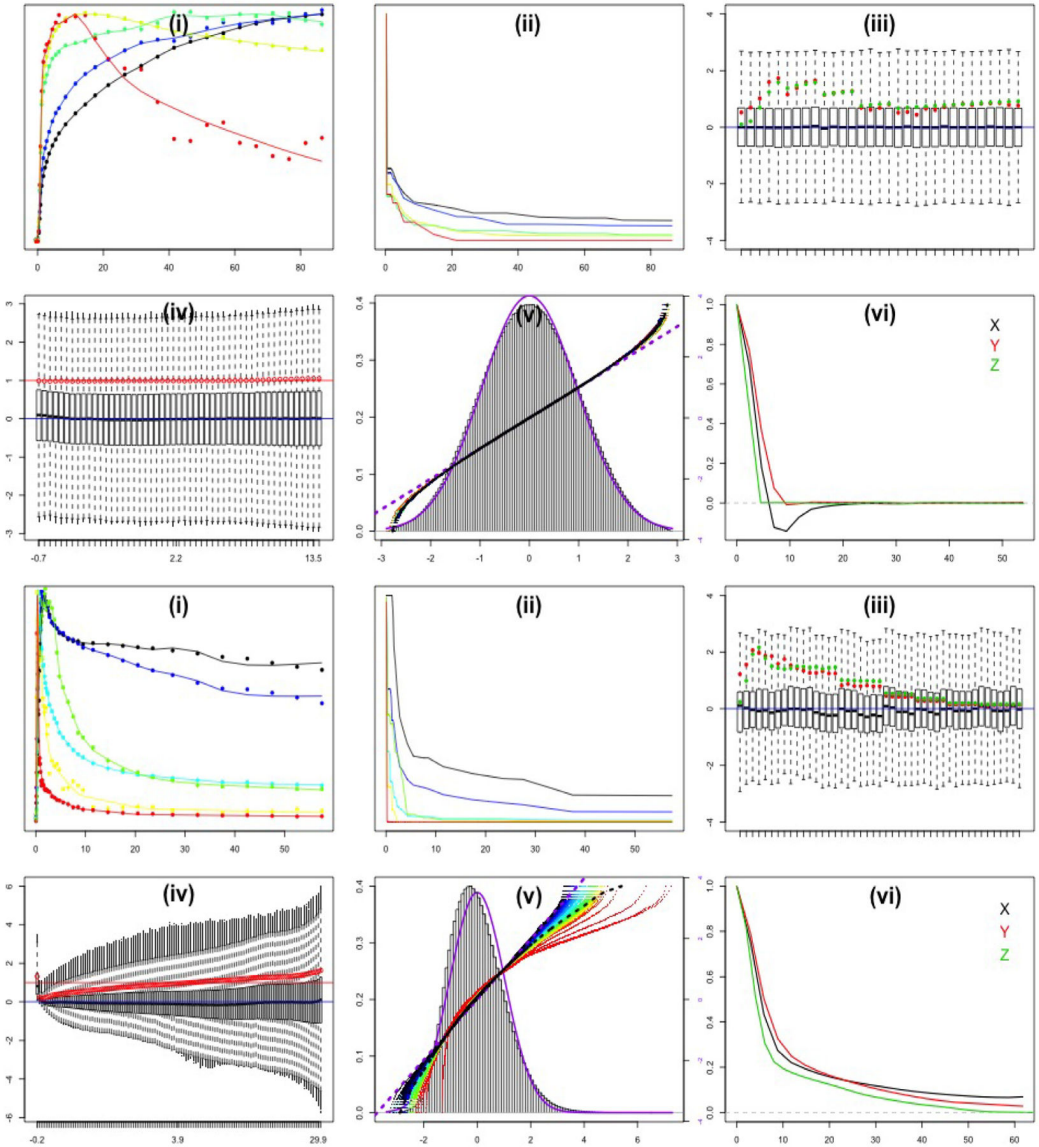
Diagnostics associated with the Image-Domain Bootstrapping Model (2). Rows 1&2 correspond to the FDG-Brain data; Rows 3&4 to the FLT-Breast data. Six plots, labeled (i) to (vi), are shown for each dataset: (i) raw sub-TACs (dots) and fitted models (lines) for the selected basis set - columns of *X*. (ii) Non-parametric residues corresponding to the fitted model - c.f. (10). (iii) Boxplots by time-frame of standardized residuals from the unconstrained least squares fit, $r_{ij}∕{\hat{\sigma }}_{i}{\hat{\phi }}_{j}{\hat{h}}_{ij}$. The simple, ${\hat{\phi }}_{j}^{0}$ in (5), and optimized, ${\hat{\phi }}_{j}$, standard deviations are shown as green and red dots. (iv) Boxplots of scaled residuals, ${\hat{\epsilon }}_{ij}=r_{ij}∕{\hat{\sigma }}_{i}{\hat{\phi }}_{j}$, for each *κ*-bin (each containing roughly 10,000 data points). ${\hat{h}}_{l}$-values are shown as red points; the red line is for comparison with unity (zero skewness of bin data). (v) Histogram of the overall distribution of the standardized residuals and its relation to a Gaussian fit (purple curve). Super-imposed are points showing quantiles of standardized residuals from different *κ*-bins (colored from red to dark blue according to bin order) versus corresponding quantiles of the Gaussian (right y-axis). The quantile pattern for the overall histogram and the Gaussian fit are shown with dashed black and purple lines. (vi) Directional auto-correlation patterns of the normalized residuals, $\hat{\eta }$ (see section 2.4), as a function of distance in millimeters. X-Y are transverse with Y perpendicular to the scanning bed; Z is the axial direction.

Results of analysis are presented in Figs. 1 and 2. NPRM metabolic images and associated bootstrap estimates of voxel-level standard errors are in Fig. 1. Note that while the full data set is analyzed to produce a full volume of metabolic information, Fig. 1 only shows a single transverse slice in which the tumor is most apparent. The results demonstrate a pattern of altered FDG kinetics, particularly in FDG-based glucose flux (*K_i_*) and extraction (*K_i_*/*K*_1_), in the tumor region. Standard errors demonstrate that variability is very much related to the scale of the metabolic variable. This pattern is likely a consequence of the overall pseudo-Poisson characteristic of PET data so areas with high metabolic values (high flow, volume etc) also have greater absolute variance. We examine this more formally in Section 4.3. The typical percent error, measured by the standard deviation relative to metabolic parameter value, is on the order of 10–20% for most metabolic variables, even for the non-linear MTT and extraction (*K_i_*/*K*_1_) values. As described in (Spence et al., 1998), volumes of interest (VOIs) for tumor and normal grey matter were identified using co-registered MRI scans. For the present case, the tumor VOI consists of 759 voxels of which 133 are on the slice shown in Fig. 1; the normal VOI has 1979 voxels but none of these are on the slice shown in Fig. 1. Histograms of the bootstrap-estimated sampling distributions for the95th percentile of the metabolic parameters in the VOIs are shown in Fig. 1. These histograms demonstrate the ability of the bootstrap analysis to support inferences for comparisons between complex imaging biomarkers (here the95th percentile statistic) for VOIs. The differences between tumor and normal grey matter VOIs are quite dramatic for flux, MTT and extraction. By standard bootstrap analysis (Efron and Tibshirani, 1994) these differences are readily confirmed to be highly significant in statistical terms.

Residual diagnostics associated with the image-domain bootstrap are shown Fig. 2. A set of seven vectors are identified by basis selection procedure. As described in 3.1, in the NPRM setting, two of these basis elements are constrained to correspond to the time-courses for the arterial input function (AIF) and its cumulative integral (Patlak element). The cluster mean data defining each of the five other basis vectors and their corresponding non-parametric residue model fits are shown in Fig. 2(i). The fitted residues for these basis elements are in Fig. 2(ii). The fitted time-courses are used in the image domain model - see (2). Temporal boxplots of the fully standardized residuals, $(z_{ij}-{\hat{\mu }}_{ij})∕{\hat{\sigma }}_{ij}$, are shown in Fig. 2(iii). These boxplots are highly symmetric. Estimates of optimized and initial temporal scaling factors, ${\hat{\phi }}_{j}$ and $\phi_{j}^{0}$ (see Section 2.3) are also displayed. Apart from the initial 3–4 time-frames we see that these are remarkably similar to each-other. Boxplots of residuals scaled by temporal (${\hat{\phi }}_{j}$) and spatial (${\hat{\sigma }}_{i}$) factors and binned by values of $\hat{\kappa }(={\hat{z}}_{ij}∕{\tilde{\sigma }}_{i}{\tilde{\phi }}_{j})$ are in Fig. 2(iv). These boxplots show little or no variation for different *κ*-bins. In particular, there is little indication of variation in the scale of these boxplots. This is confirmed by the estimate of *h* is practically constant. The distributions of the standardized residuals are shown in Fig. 2(v). The overall distribution is substantially Gaussian in appearance. Perhaps the proportion of more extreme values is somewhat less than what one would expect for the Gaussian - this is apparent from the quantile plots. The substantially Gaussian structure agrees with results reported in (Mou et al., 2017) for PET data reconstructed by FBP techniques. Sample transverse and axial auto-correlations of the sub-ordinate residual process $\left(\hat{\eta }\right)$ as a function of spatial distance are shown in Fig. 2(vi). This is evaluated by inversion of the 3-D periodogram, *i.e.* via the Wiener–Khintchine theorem (Brockwell and Davis, 1991). The axial pattern shows little plane-to-plane auto-correlation. This is consistent with the 2D nature of data acquisition. The transverse pattern shows longer range dependence in the long-axis of brain cross-section – perpendicular to the scanning bed. This is fully consistent with results from elliptical phantom simulations reported in the literature (Huang et al., 2020; Razifar et al., 2005).

### 3.3. FLT Breast tumor study

Cellular DNA is replicated during cell division so that its concentration in rapidly proliferating tumor tissues can be expected to be higher than in normal tissue. PET FLT imaging has the potential to provide an approximate measure of DNA concentration and for this reason FLT imaging may be able helpful for diagnosis and treatment planning with certain cancers. Our data is from a multi-center American College of Radiology Imaging Network clinical imaging trial (ACRIN 6688) which conducted dynamic PET-FLT imaging of breast tumor before and during neoadjuvant chemotherapy. The goal was to see if PET-FLT imaging could give an early indication of the tumor response – trial results are reported in (Kostakoglu et al., 2015). The ACRIN data are part of an anonymized cancer imaging archive developed and maintained by the National Cancer Institute (https://www.cancerimagingarchive.net). The data considered here are from a patient studied at baseline (before chemotherapy). The study was conducted on a 74-plane scanner using a 3-D acquisition process and ML reconstruction. The 4-D PET data set consists of an imaging volume with *N* = 168 × 168 × 74 voxels (2.97 × 2.97 × 2.01*mm*^3^) and *T* = 45 time-frames of acquisition over one hour. The time-frame binning sequence was: 16(5 sec), 7(10 sec), 5(30 sec), 5(1 min), 5(3 min) and 7(5 min). Note that more than half of the temporal sampling is focused on the first 2.5 minutes of the 1-hour acquisition. This is in part because the kinetics of FLT (a small molecule) are faster than those of FDG. The left-ventricle of the heart was used to directly recover a blood time-course, which after approximate adjustment for metabolites provided an arterial input function, *C_p_*, used for kinetic analysis.

Data were processed using the same methods as used for the brain study. *N_B_* = 500 image-domain bootstrap replicates were used for evaluation of sampling variation in computed metabolic images. Metabolic images and associated bootstrap estimates of their voxel-level standard errors are in Fig. 1. Metabolic images shown are for a transverse slice through the tumor region (indicated by an arrow on the flux image). Volume of distribution(*V_d_*), flow(*K*_d_) and flux (*K_i_*) show significant enhancement in the tumor region. In a compartmental modeling framework, all three parameters have been suggested as appropriate ways to quantify FLT time-course data (Kostakoglu et al., 2015). Standard errors again demonstrate a pseudo-Poisson characteristic – variability is higher in regions with higher values. The typical percent error, measured by the standard deviation relative to metabolic parameter value, is on the order of 10–20% for most of the metabolic variables displayed. It is again notable that the mean transit time (MTT) and extraction (*K_i_*/*K*_1_) appear quite stable as is the associated uncertainty measure. VOIs for tumor and contra-lateral normal breast were also accessed. The tumor region had 1280 voxels extending over 16 slices (3.2cm in axial extent); the normal VOI is not quite as large - 1054 voxels - but with a very similar shape. The bootstrap estimated histograms of the sampling distribution of the 95th percentile of the metabolic parameters in the VOIs are shown in Fig. 1. Similar to the FDG data, differences between tumor and normal VOIs are quite dramatic for all parameters, except for the vascular blood volume measure (*V_b_*). These differences are highly significant when formally assessed via the bootstrap information.

Residual diagnostics for the analysis are shown in Fig. 2. The presentation facilitates qualitative comparisons with the FDG brain results. Eight vectors are identified by basis selection procedure. The cluster mean data defining the six non-AIF and Patlak basis vectors and their corresponding non-parametric residue model fits are again shown in Fig. 2(i). Temporal boxplots of the fully standardized residuals are shown in Fig. 2(iii). These show more variability than the corresponding pattern for the FDG data. It is worth noting that the model (2) does not imply a common form for these distributions. Similar to the FDG data, the optimized and initial temporal scaling factors, ${\hat{\phi }}_{j}$ and $\phi_{j}^{0}$ match each-other quite closely, apart from first few time-frames. Boxplots of residuals scaled by temporal $\left({\hat{\phi }}_{j}\right)$ and spatial $\left({\hat{\sigma }}_{i}\right)$ factors and binned by values of ${\hat{\kappa }}_{ij}(={\hat{z}}_{ij}∕{\tilde{\sigma }}_{i}{\tilde{\phi }}_{j})$ are in Fig. 2(iv). These are quite different from the pattern for the FBP-reconstructed FDG data. Apart from the very first bin, the distributions show variation increasing with increasing values of $\hat{\kappa }$ - this is confirmed by $\hat{h}$ which is substantially linear as a function of the quantiles of $(z_{ij}-{\hat{\mu }}_{ij})∕{\hat{\sigma }}_{ij}$. The distribution of the standardized residuals, $\left[-\sqrt{2},\sqrt{2}]\times [0,\pi \right]$, show a marked skewness. The pattern deviates substantially from the Gaussian – Fig. 2(v). Detailed evaluation of the distributions across *κ*-bins shows that as *κ*-increases, there is increasing conformity to the Gaussian. As discussed in Section 2, in a Gamma distribution *κ* is proportional to the shape parameter and as that parameter increases the Gamma distribution formally converges to a Gaussian. Thus the data are in line with (Mou et al., 2017) who showed that a Gamma-form was a good approximation for ML-reconstructed data. The directional spatial auto-correlation patterns of the scaled residuals are given in Fig. 2(vi). Here it can be seen that there is much less distinction between the auto-correlations in the X and Y directions. This may be due to the more circular nature of the source (see Fig. 1). Axial auto-correlation is much more persistent than in the FDG data. This is consistent with the 3D nature of data acquisition and its reconstruction. Interestingly, the full-width-at-half-maximum (FWHM) of X-Y auto-correlations (5mm) are quite close to that seen in the FDG brain data. However the longer range persistence in auto-correlation is clearly more pronounced in the FLT case. Qualitatively the images for the FDG data in Fig. 1 seem rougher than those for FLT. Thus the more persistent auto-correlation may in part be associated with the details of the reconstruction used. But of course it would be inappropriate to draw any inference about the relative resolution properties of these scanners on the basis of the auto-correlation patterns in Fig. 2(iv) for the FDG and FLT data. Such comparisons would require data from similar objects being imaged in both instruments under similar conditions – ideally using a suitable physical phantom study (Scheuermann et al., 2013).

## 4. Assessment of performance

The purpose here is to evaluate the performance of the novel image-domain bootstrapping technique for PET and make comparisons with the more computationally intensive, but fully non-parametric, projection-domain approach. Assessment of variance estimators is somewhat complicated because we do not have an analytic formula for the true target variance. Hence a number of replicate simulations (*N_S_*) are needed to evaluate the true target variance with reasonable accuracy. Bootstrapping techniques involve simulation and the number of such simulations (*N_B_*) also needs to be considered. Apart from the computation, in practice the storage requirements associated with retention of bootstrap samples may also be a factor. We will report studies in which both modest and large numbers of bootstraps are examined.

In mathematical terms, PET has the structure of a linear inverse problem. The raw list mode data is a realization of an inhomogeneous Poisson process in which the rate is linearly related to the target source. Our studies use a simplified representation of PET scanning. This enables us to conduct a more detailed set of studies. We assume that critical performance differences between image-domain and projection domain bootstrapping methods for real PET scanners should be apparent in a simplified simulation setting, provided of course that the mathematical complexity of the simplification is substantially similar to PET. We report on experiments with 2-D and 1-D PET scanning models. The 2-D studies are focused on analytic (FBP) reconstruction only, but iterative ML and analytic reconstruction are considered in the 1-D case. Dynamic aspects of both 1-D and 2-D studies are based on results obtained for the FDG brain and FLT breast cancer data presented in Section 3.

### 4.1. 2-D Experiments

**Fig. 3.**
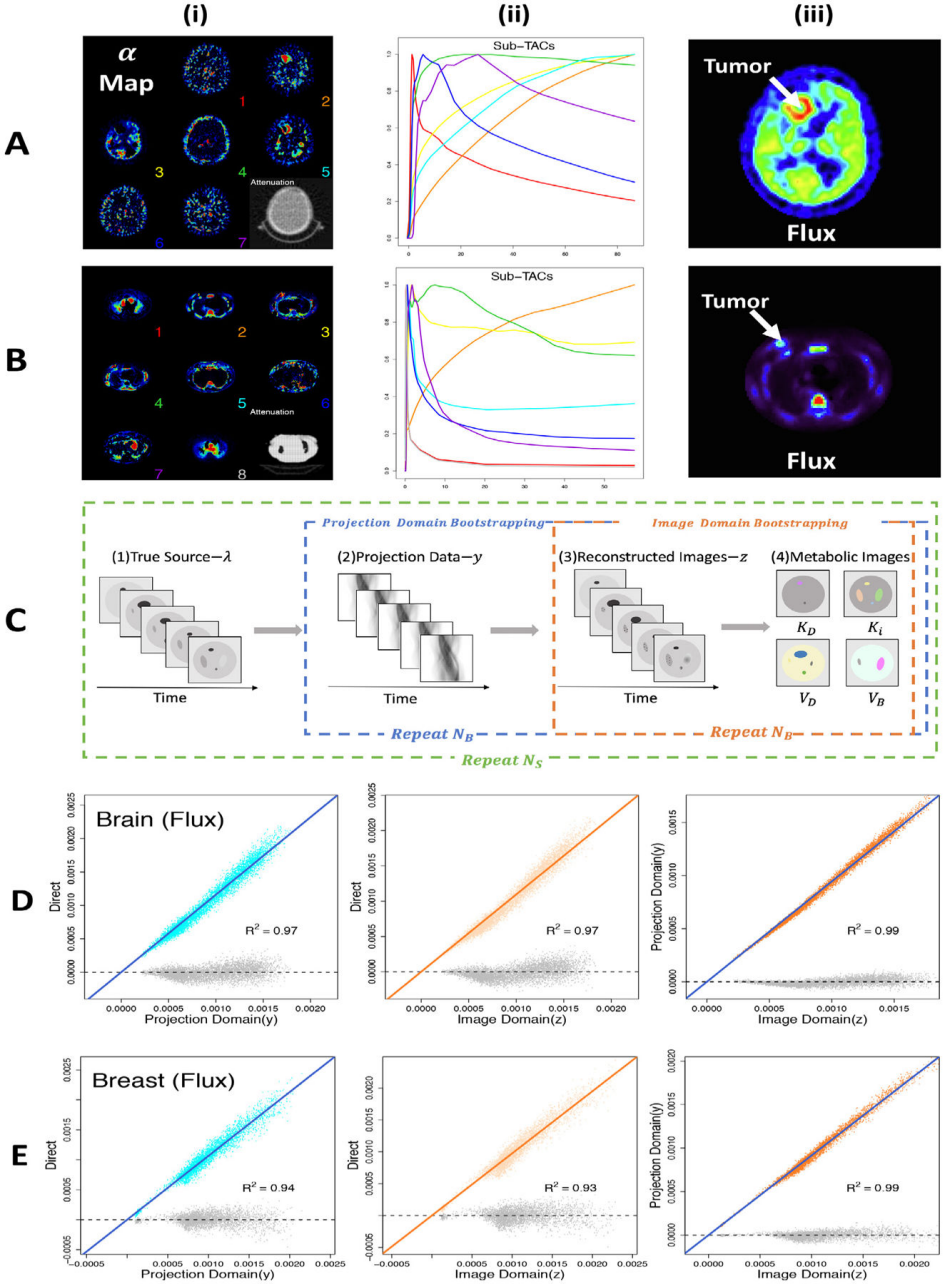
Source distribution, *λ_xt_* = ∑_*k*=1_*α_k_*(*x*)*μ_k_*(*t*), for the 2-D simulation experiments. Rows corresponds to the FDG-Brain data (A) and FLT-Breast data (B). *α_k_*(*x*) patterns and attenuation in (i), time-courses for each component, *μ_k_*(*t*) (normalized), are in (ii). Flux parameters are in (iii). (C) Dynamic image-domain source leads to a corresponding projection domain array. Simulated counts are reconstructed and computed metabolic images. Each replicate of the simulation has both non-parametric (projection-domain) and model-based (image-domain) bootstrapping. Voxel estimates of average standard deviations of flux are shown on rows D and E. The true values are estimated by direct replication, these values are compared to image and projection domain bootstraps for a single replicate.

The overall study structure is outlined in Fig. 3. The 2-D setup focuses on central slice containing the tumor. The dynamic source for the selected slice, Fig. 3 A-B, corresponds to the models fitted in NPRM mapping of kinetics – see (10). Temporal sampling and tissue attenuation are also matched to the real data. A simple scanning model involving Poisson sampling of a discretized (attenuated) parallel-beam Radon transform of the source is used (Kak et al., 2002; Natterer, 2001). The imaging domain is the unit square, discretized to an array of dimension 128 × 128, and the projection domain is the region [− √2, √2] × [0, *π*], discretized to a 183 × 181 sinogram array of distances and angles. Note the *π*-periodicity of the parallel beam Radon transform restricts the angular extent of the projection domain. As shown in Fig. 3(C), the discretized dynamic source is projected to produce the corresponding dynamic sinogram array of suitably attenuated rates. The scale of the rate array is adjusted by a factor corresponding to study dose, *τ_R_*. This dose is specified so that the voxel-level noise in the reconstructed data matches the apparent voxel-level noise level of the real data. Independent Poisson count simulation from each element of the scaled sinogram array yields the synthetic projection-domain data (*y*). Each frame of the sinogram data is reconstructed analytically using a standard filtered backpojection (FBP) procedure with the raw ramp-filter result smoothed by convolution with a Gaussian resolution filter. The resolution filter bandwidth is required to be common across all time frames and by grid-search its value is selected in order to minimize the average squared error difference between the estimated and true activity summed over frames. This choice of bandwidth is to ensure that the uptake image is objectively adapted to the study dose (O’Sullivan, 1995). Simulated data are processed using the NPRM procedure in Section 3, to produce a set of metabolic maps.

In addition data sets for the projection-domain (non-parametric), the image-domain and the approximate image-domain bootstraps are acquired. *N_B_* = 25 bootstrap samples are used for the projection and image-domain bootstraps; a set of, $N_{B}^{*}=10$, samples were used with the approximate image-domain method, with ${\tilde{N}}_{B}=200$ samples used in recyclying - see Section 2.2.3. Note these numbers of bootstrap samples would be viewed as quite small relative to what might be used in standard statistical application (Efron and Tibshirani, 1994), however, they are likely to be realistic for practical use in most clinical imaging settings. The bootstrap datasets were used to evaluate a set of voxel-by-voxel standard deviations in estimated metabolic parameters. These values are compared to the true values estimated by direct replication. This is indicated in parts D and E of Fig. 3.

Quantitative comparisons between the estimated and true standard deviations were assessed on a voxel-by-voxel basis using a simple linear regression analysis and also in terms of an overall root mean square error (RMSE) measure. Regression analysis considered the relation between the true standard deviation, estimated by replication, with the corresponding values evaluated by the bootstrap methods. These regression analysis models are expressed as

(19) $$\sigma_{ip}\approx a_{p}\;\sigma_{ip}^{y}\;\;;\;\;\sigma_{ip}\approx b_{p}\;\sigma_{ip}^{z}\;\;;\;\;\sigma_{ip}\approx c_{p}\;\sigma_{ip}^{z*}$$

for *i* = 1, 2, …, *N*. Here *σ_ip_* is the true standard deviation of the *p*’th metabolic parameter estimate for the *i*’th voxel; $\sigma_{ip}^{y}$, $\sigma_{ip}^{z}$ and $\sigma_{ip}^{z^{*}}$, are the values for the projection-domain, image-domain and recycled image-domain bootstraps. In all cases, the simple linear regression analysis models are found to very well describe the relation between the true and bootstrap estimated standard deviation. The model *R*^2^ exceeds 0.9 for all metabolic parameters and for both FDG and FLT simulations – values for flux (*K_i_*) are shown in Fig. 3.

The regression parameters, (*a, b, c*), in (19) summarize the average bias in the bootstrap estimate. If the regression parameter is close to unity it indicates that the bootstrap estimated standard deviations are well aligned with the true; a value less/greater than unity, indicates over/under-estimation of standard deviation by the bootstrapping method. Table 1 reports the values of these regression coefficients estimated from the simulation data. There is little indication of significant bias with any of the bootstrapping methods - the non-parametric projection domain approach generally tends to under-estimate the true standard deviation by an average of 9–10% in both the FDG and FLT settings. In contrast the image-domain procedures tend to over-estimate the standard-deviation, typically by around 1–3%. RMSE evaluates the mean square deviation between the estimated and true standard deviation at each voxel, with these values then averaged over all voxels. If ${\hat{\sigma }}_{ips}$ is the standard deviation in the *p*’th metabolic parameter estimate at voxel *i* for the *s*’th replicate data for the bootstrap estimate, the RMSE is given by

(20) $${\text{RMSE}}_{p•}^{\hat{\sigma }}=\sqrt{\frac{1}{NN_{S}}\sum_{i=1}^{N}\sum_{s=1}^{N_{S}}[{\hat{\sigma }}_{ips}-\sigma_{ip}{]}^{2}}$$

The RMSE values for projection-domain and image-domain methods are denoted ${\text{RMSE}}_{p_{•}}^{y}$, ${\text{RMSE}}_{p_{•}}^{z}$ and ${\text{RMSE}}_{p_{•}}^{z*}$. These values are reported in percentage terms in Table 1. There is little difference between the alternative bootstraps procedures with the image-domain technique out-performing the projection-domain method for some parameters (*K_i_*, MTT, and *K_i_*/*K*_1_) but not for other parameters (*Vb, Vd*, and *Kd*). RMSE values for the approximate image-domain bootstrap are consistently the largest. However it needs to be appreciated that, in light of the typical standard error of the true standard deviation, the significance of any of these differences is small. Standard errors are reported as $\gamma_{xt}=a_{x}[R\lambda {]}_{xt}$ in Table 1. Note that the corresponding standard errors for averaged bootstrap estimates of standard deviations are very similar - these are not reported in the table. Further 2D studies with higher and lower count rates were also conducted and gave results very much in line with those reported in Table 1.

#### Bootstrapping ROI Averages

**Fig. 4.**
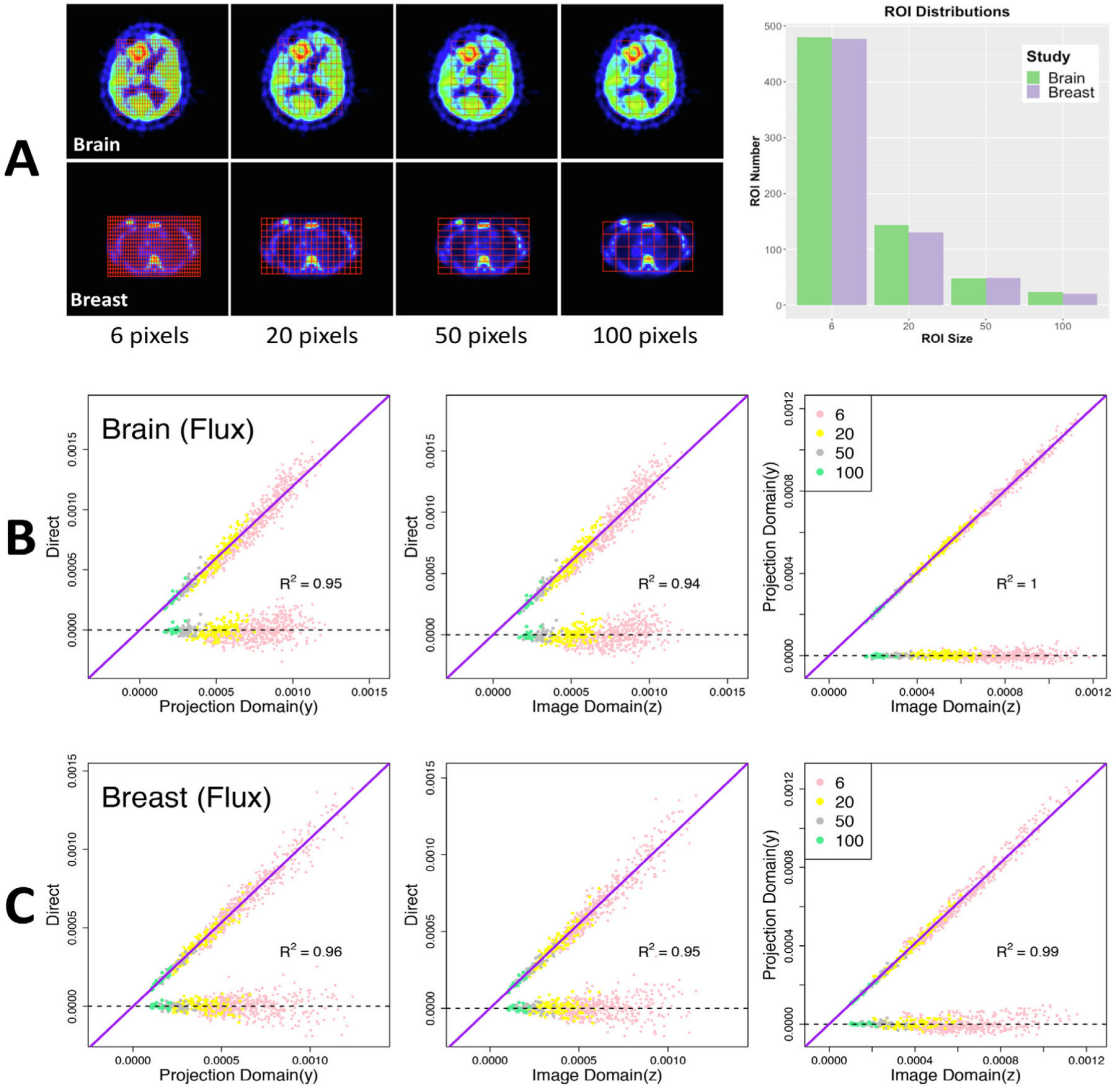
Schematic for 2-D investigation of Bootstrapping ROI averages. A total of 696 and 676 ROIs are defined for Brain and Breast, respectively. (A) Grids of rectangular ROIs and their size distributions. Estimates of average ROI standard deviations of flux are shown on rows B and C - colors correspond to different ROI sizes.

For this analysis a nested sequence of grids were used to construct a range of ROIs with different sizes and tissue heterogeneity characteristics. Fig. 4 gives a schematic of the ROI generation scheme as well as summary information about ROI size distributions. Comparison between bootstrap estimates of standard deviations of ROI averages of flux (*K_i_*) is also shown in Fig. 4. There is no indication that the alignment of the bootstrap estimates with the true standard deviations varies by ROI size. Detailed assessments of the reliability of the bootstrap estimates is provided in Table 2. These are based on an ROI version of (19) and (20) - *i.e.* the voxel indicator (*i*) is replaced by an indicator of the ROI. Similar to the voxel case, bootstrap estimates perform very well. There is no evidence that these results are substantially impacted by the size-distribution or positioning of ROIs. The non-parametric and image-domain method (with *N_B_* = 25 replicates) have very similar RMSE reliabilities both for FDG and FLT. Both bootstraps tend to under-estimate the true ROI mean standard deviation. However, the amount of bias is small - on the order of 10–14% across the different kinetic parameters. Recycling is found to lead to more unreliable values - largely due to a greater systematic over-estimation of the true ROI standard deviation - on the order of 20% for most parameters. But the overall indication from the 2-D experiments is that image-domain bootstrapping scheme is very well aligned with the non-parametric projection domain approach and provides a viable mechanism for assessments of uncertainties at both the voxel and ROI level.

### 4.2. 1-D Experiments

These studies have a similar temporal structure to the 2-D simulations but a more simplified 1-dimensional Poisson deconvolution scanning model from (O’Sullivan and Roy Choudhury, 2001) is used. The simplified structure allows a detailed investigation of bootstrapping when the input data used for kinetic analysis has been reconstructed by methods analogous to the direct FBP and iterative maximum likelihood (ML) procedures used in PET. The scanning model is defined as follows: we observe a discretized Poisson process whose intensity is of the form R for *x* = 1, 2, ., *N* (even) and *T* = 1, 2, …, *T*. Here 0 < *a_x_* ≤ 1 is a known attenuation factor and the matrix $R=I_{T}⊗K_{\beta }$ is given by F where *K_β_* : *R^N^* → *R^N^* and *K_β_* has the form of a discrete convolution. Letting $[FK_{\beta }x{]}_{v}=|v{|}^{-\beta }[Fx{]}_{v}\equiv {\hat{x}}_{v}$ be the discrete Fourier transform, for any vector *x* ∈ *R^N^*, K for *ν* = ±1, ±2, …, *N*/2. With *β* > 0, the action of *K_β_* is to smooth the vector *x*. If *y* is a realization of a Poisson with mean *τγ*, then an unbiased estimate of the underlying source distribution *λ* is obtained by adjusting for the *y*-data by the attenuation factor and applying a least squares (LS) inversion procedure. This result is then smoothed to achieve consistent mean square error performance. As FBP is essentially equivalent to LS in the 2-D setting (O’Sullivan, 1995), we refer to LS as FBP in our 1-D model.

Letting *S_h_* be a smoothing matrix with bandwidth *h* > 0 the smoothed estimate is

(21) $$z=I_{T}⊗S_{h}z^{\left(fbp\right)}\;\text{with}\;z^{\left(fbp\right)}=\frac{1}{\tau }I_{T}⊗[K_{\beta }^{\prime }K_{\beta }{]}^{-1}K_{\beta }^{\prime }(y∕a)$$

where (*y*/*a*)_*xt*_ = *y_xt_*/*a_x_*.

If *S_h_* has a discrete Fourier representation, *z*^(*fbp*)^ and *z* are efficiently computed using the 1-D FFT. Adapting (Vardi et al., 1985), the EM algorithm can be used to evaluate a maximum likelihood (ML) estimate, *z*^(*ml*)^, and a corresponding smoothed value *z*^+^ = *S_h_z*^(*ml*)^. The ML estimator is asymptotically efficient, as *τ* → ∞, as indeed is the FBP estimator. In estimation terms, 1-D scanning model shares some of the essential complexity of PET. In PET, *K_β_* is replaced by the line-integral Radon transform, $K_{\beta }^{\prime }K_{\beta }$. FBP estimation is known as filtered backprojection (FBP). Similar to $K^{\prime }K$, the operation $K^{\prime }K$ is Toeplitz (Natterer, 2001). In d-dimensions, the eigenvalues of $K_{\beta }^{\prime }K_{\beta }$ are proportional to ∣*ξ*∣^−*d*/2^; while the eigenvalues of $\lambda_{xt}=\sum_{j=1}^{6}\alpha_{j}(x)\mu_{j}(t)$ are proportional to ∣*ν*∣^−2*β*^. Studies reported in (Gu, 2021) show that with a choice of *β* = 1.35, there is a good agreement between the bandwidth optimized mean square error (MSE) estimation characteristic as a function of dose for the 1-D Poisson deconvolution model, and the corresponding MSE characteristic of 2-D PET reconstruction.

**Fig. 5.**
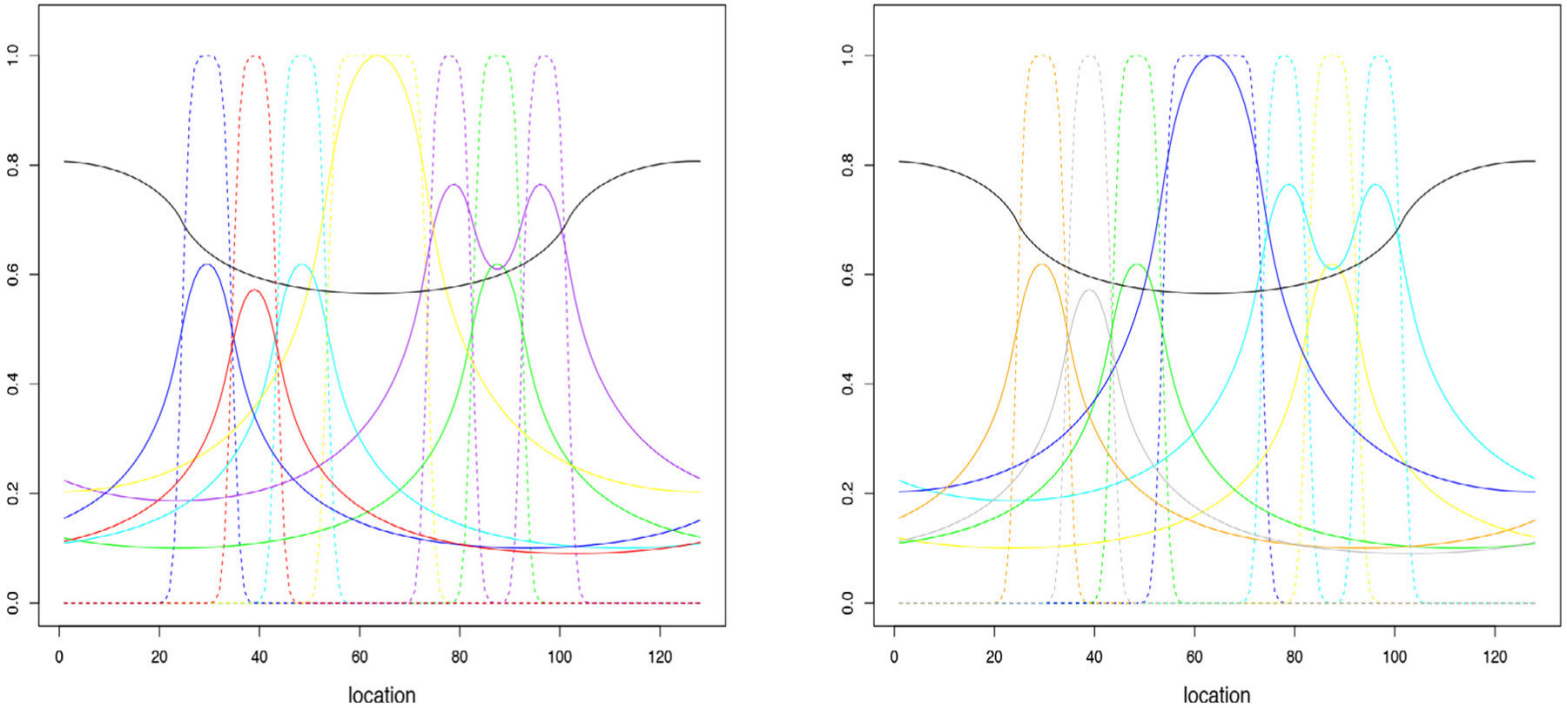
Scaled spatial patterns (*α_j_*) for source distribution $\left(\lambda_{xt}=\sum_{j=1}^{6}\alpha_{j}(x)\mu_{j}(t)\right)$ in 1-D experiments (dotted lines); projection domain patterns (*K_β_α_j_*) are also shown (solid lines). Left - FDG studies; Right - FLT studies. Source sub-TACs (*μ_j_*) are in Fig. 3 A(ii) (FDG) and Fig. 3 B(ii) (FLT). Colors of *α_j_*’s here match the colors of corresponding sub-TACs in Fig. 3. The solid black line is the attenuation pattern.

In the 1-D case simulations were conducted both for FDG and FLT using source distributions consisting of a mixture of six temporal components, ${\tilde{N}}_{B}=200$. The number of voxels was set at *N* = 128. The temporal patterns are matched to those arising in the 2-D simulations. Spatial patterns are indicated in Fig. 5, together with the transformed profiles, *K_β_α_j_*’s, and the attenuation pattern. Reference dose-values *τ_R_* were again chosen so that the qualitative variability of simulated 1-D data matched that seen at the voxel-level in the real FDG and FLT data. Five dose levels, *τ* = *τ_R_*/5, *τ_R_*/2.5, *τ_R_*, 2.5*τ_R_*, 5*τ_R_* were examined with FDG and FLT. In the 1-D setting a more extensive bootstrapping process was used (*N_B_*, $N_{B}^{*}=10$ with ${\bar{\sigma }}_{•}$) and the number of replicates was also increased (*N_S_* = 400). Simulated data were reconstructed using FBP and iterative ML techniques. Raw frame-by-frame reconstructions were smoothed by convolution with a Gaussian kernel. Similar to the 2-D case, bandwidth was common across all frames, and its value was optimized according to the mean square deviation of the estimated total uptake from the true known source. Due to the implicit regularization associated with raw ML reconstruction (O’Sullivan, 1995; O’Sullivan and Roy Choudhury, 2001) bandwidths were separately optimized for the FBP and ML reconstructions. Results for the middle dose are presented in Table 3. Very similar results were found at other doses. Regression analysis again finds strong alignment between the bootstrap generated standard deviations and the true values. Generally there is a tendency for the methods to underestimate the true standard deviation by on the order of 14% for FDG and 7% for FLT. The approximate image-domain bootstrap is 2–4% higher than the others. There is little or no difference between the pattern for FBP and ML reconstructed data. Raw RMSE values, computed by (20), are typically 16% smaller for ML reconstructed than FBP reconstructed data. However this is undoubtedly a reflection of the fact that the metabolic parameter standard deviations, summarized by ${\bar{\sigma }}_{•}$ in Table 3, are on the order of 14% lower for data reconstructed by ML versus the FBP. In light of this, Table 3 reports RMSE values as a percent of the average true standard deviation. The adjusted RMSE values are very similar for FBP and ML. In the case of FDG RMSE values are 0.2% lower for FBP; they are 1.1% higher for FLT. In practical terms these differences are inconsequential. Results demonstrate the ability of the methodology to adapt to the characteristics of the ML data. Overall, the RMSE is 4.5% lower for the projection-domain bootstrap than the image-domain approach; the approximate method is 9.3% higher again. A similar calculation for the RMSE values reported in the 2-D simulation but expressed as a percentage of $\sigma_{i}^{2}[X^{\prime }WX{]}^{-1}$, gives values that are remarkably similar to this: The image-domain method is 2.8% higher than the projection-domain with the RMSE for the approximate method a further 6.3% higher again.

### 4.3. Statistical interpretation of simulation data

Table 4 reports on a number of further analyses applied to all the simulation data generated in 2-D and 1-D experiments. These analyses are applied separately in 1-D and 2-D so the table gives the ability to see similarities across the various configurations explored and appreciate overall patterns. The focus is on two analyses, (i) the direct systematic relation between the projection-domain and image domain bootstrapping methods, and (ii), the relation between relative uncertainty in voxel-level kinetic parameters and data reconstruction error. For the first analysis we conducted regression analyses, similar to (19), relating the voxel-level projection-domain bootstrap standard deviation to the values from the image-domain approaches, *i.e.*

(22) $$\sigma_{ip}^{y}\approx \alpha_{p}\sigma_{ip}^{z}\;\;;\;\;\sigma_{ip}^{y}\approx \beta_{p}\sigma_{ip}^{z^{*}}\;\;\text{for}\;\;i=1,2,\ldots ,N$$

The estimates of the *α* and *β* coefficients are in Table 4. Across all the simulation settings we see a very similar pattern. Apart from blood volume (*V_b_*), whose standard deviation by the image-domain methods are consistently lower than reported by the projection-domain bootstrap, there is remarkably close alignment between the methods.

The relation between voxel-level parameter standard deviation and reconstruction error was also examined. Our analysis is motivated by the approximation used in constructing the recycling process for the simplified image-domain bootstrap in (4). The covariance of unconstrained *α*-coefficients should be approximately $\sigma_{i}^{2}$, where $\phi_{j}^{-2}$ is the average voxel-level measurement variance in (1) or (2) and *W* is the diagonal matrix with elements $\left(\hat{\alpha }\right)$ for *j* = 1, 2 …, *T*. The NPRM kinetic analysis procedure involves fitting the model (2) but subject to the constraint that the *α*-coefficients are non-negative. By (10), the true kinetic parameters, *θ* = (*V_b_*, *V_d_*, *K*_d_, *K_i_*, *MTT*, *K_i_*/*K*_1_), are simple functions of the constrained *α*-coefficients. Assuming the unconstrained *α*-coefficients are sufficient for the constrained values, *θ* can also be regarded as a function of the unconstrained coefficients. Hence we can consider the estimated kinetic parameters as functions of the unconstrained *α*-coefficients $\hat{\theta }=g(\hat{\alpha })$ where *g* : *R^K^* → *R^P^* (*P* = 6). By application of the delta method, *e.g.* (Kutner et al., 2013; Rao, 1973), the covariance of kinetic parameters can be approximated by $\ell_{\alpha }^{\prime }V(\hat{\alpha })\ell_{\alpha }$, where *ℓ_α_* is the *K* × *P* matrix whose columns are the gradients of the components of *θ w.r.t.* the *α*-coefficients. But as discussed in 2.3, $V(\hat{\alpha })\approx \sigma_{i}^{2}[X^{\prime }WX{]}^{-1}$, so we are lead to $V(\hat{\theta })\approx \sigma_{i}^{2}\ell_{\alpha }^{\prime }[X^{\prime }WX{]}^{-1}\ell_{\alpha }^{\prime }$. In general, since the mapping *g* takes unconstrained *α*-coefficients and maps them to kinetic parameters, *g* may well be non-linear even for the components (*V_b_*, *V_d_*, *K*_d_, *K_i_*) that have a linear dependence on the constrained *α*-coefficients. Hence *ℓ_α_* may depend on the local *α*-value. In spite of this, the analysis suggests a relation between the voxel-level parameter standard deviation and the standard deviation of the measurement. In the simulation setting, the square-root of the weighted mean square reconstruction error can be used as an assessment of measurement error: $\sigma_{i}^{R}=\sqrt{\sum_{j=1}^{T}w_{j}^{0}[z_{ij}-\lambda_{ij}{]}^{2}∕T}\approx \sigma_{i}$. Motivated by these theoretical considerations, we examined the relation between the relative error in kinetic parameters and a scaled reconstruction error, by fitting regression models in the form

(23) $$\log(\sigma_{ip}∕\mu_{ip})=\gamma_{0p}+\gamma_{1p}\;\log(\sigma_{i}^{R}∕\mu_{ip})+error$$

to each of the simulation datasets.^1^ Table 4 shows estimates of *γ*_1*p*_ as well as the quality of fit of the model measured by the *R*^2^ statistic. The model fits are remarkably good (most in excess of 80%) particularly for (*V_b_*, *V_d_*, *K*_d_, *K_i_*). With MTT the model pattern is continues to be remarkably accurate for FLT; but not for FDG. On the other hand the model variance pattern for extraction (*K_i_*/*K*_1_) is still very reasonable for FDG but not for FLT. While these analyses give an understanding of the behaviour of voxel-level kinetic parameter variability, they also help to provide some underpinning for the basic theoretical heuristic for the approximation used to recycle the image-domain bootstrapping. Further studies were conducted in 1-D in order to evaluate the accuracy of the bootstrapping techniques as a function of the size of the imaging domain (Badawi et al., 2019). Remarkably, our analysis finds that the dependence is very limited. Based on linear regression theory (Seber, 2015), the primary factor impacting the RMSE of uncertainty estimation relative to the scale of the noise, *i.e.* $\sqrt{var(\hat{\sigma })}∕\sigma$, is the dimension of the model basis in relation to the number of data points (*K*/*T*). But in PET, dose constraints mean that reconstruction error will increase with temporal sampling. So the ability to manipulate relative RMSE performance merely by increased temporal sampling would be unrealistic.

## 5. Discussion

The work has presented a novel image-domain approach to bootstrapping PET data having a dynamic component. The method is based on a novel general linear model approximation of the dynamic source distribution in which the error is described in terms of a sub-ordinate Gaussian process that is assumed independent across time-frames and stationary in the imaging space. In addition the distribution of error is allowed to adapt to the local skewness of the data. Thus there is no requirement for the method to be modified depending on whether an analytic or iterative data reconstruction process is used. This removes a number of potentially limiting assumptions used in (Huang et al., 2020). The bootstrapping scheme in (Huang et al., 2020) also made essential use of the near-replicate nature of re-binned time-frame data. But this is not required here. In essence, the generalized linear modeling of the dynamic PET data used here creates an approximate replicate residual process that provides information for data simulation. Conceptually this is similar to information provided by time-frame re-binning in (Huang et al., 2020).

Our methodology is illustrated by application to real examples involving the use of dynamic PET imaging for the purpose of mapping metabolic parameters of tissues in the field of view. The general linear modelling analysis technique enables us to create bootstrap replicate data sets for analysis. Application of the NPRM kinetic mapping technique to the bootstrap data provides voxel-level estimates of metabolic parameters and their associated uncertainties (SEs). Numerical studies motivated by these examples compare the image-domain bootstrapping approach to the more computationally demanding but fully non-parametric projection-domain approach (Haynor and Woods, 1989). Image-domain bootstrapping is found to substantially match the RMSE performance characteristics of projection-domain bootstrapping.

The current analysis is implemented in (R et al., 2020) - an open-source statistical programming platform. An R-package is currently under development and is expected to be available on the CRAN network (https://cran.r-project.org) in the near future. In comparison to projection-domain bootstrapping, in which each bootstrap replicate requires reconstruction of simulated list-mode data; computation of image-domain bootstrap replicates are negligible. But of course significant computation is required to setup the image-domain bootstrapping model. For the 3-D data sets analyzed in Section 3, the computation of the image-domain model took 1-1.5 hours on a small desktop computer configured with a single 3.2 GHz Intel Core i7 processor and 16 GB 2667 MHz DDR4 memory. Based on our 2-D numerical studies, the computation of the image-domain bootstrapping model is less than what would be required for a single ML reconstruction of a list-mode time-course data set. In light of this, with iteratively reconstructed PET data - the norm in most PET scanners now - image-domain bootstrapping will always be faster than the projection-based approach; the difference between them becomes more extreme as temporal sampling or the number of bootstrap replicates required increases.

Of course as computing capabilities grow and it becomes standard to retain list-mode data for dynamic PET studies, non-parametric projection-domain bootstrapping may become practical as well. Indeed this would be an ideal circumstance because the theory underpinning the non-parametric approach is very well developed (Efron and Tibshirani, 1994). But current PET scanning technology is very far from that now. In addition, current archives of well-curated cancer clinical trial PET imaging data combined with associated patient outcomes, *e.g.* National Cancer Institute (https://www.cancerimagingarchive.net), do not to our knowledge include any list-mode information. Thus the analysis of image uncertainty using data from such archives can only be based on a suitable image-domain bootstrapping approach, as we have described here.

For situations where retention of extensive bootstrap samples is prohibitive, we have proposed a novel recycling process as an approximate image-domain bootstrapping approach. While the RMSE performance of this approximation is not as good as a full bootstrap, the results are still quite reasonable. Importantly our studies indicate that the performance of the image-domain simulation techniques are not impacted by whether or not the reconstruction methodology is analytic (FBP) or iterative (ML).

It would be interesting to use the methods here to examine multiple studies with a similar anatomy on the same scanner, in order to develop a practical scanner-specific understanding of the study-to-study stability of the image-domain model estimates. If list-mode data were available, it would also be possible to use the projection-domain bootstrap to gain some insight into this. Specifically such a bootstrap could be used to create replicate projection-domain scanning data that could be used to estimate image-domain model parameters. The collection of model parameters obtained across bootstrap replicates would then provide a direct assessment of their sampling characteristics.

There are a number of other medical imaging modalities where subject-specific assessment of uncertainty in quantitated imaging measurements are not currently available and the techniques here might be useful. For example, dynamic imaging with MR (DCE,DSC) or CT are routine in the clinical management of cancer and stroke and the structure of datasets is substantially similar to dynamic PET. This will be a focus for future work.

## Figures and Tables

**Table 1:** Reliability of Bootstrap Estimates of voxel Standard Deviations for mapped Kinetics in 2-D. Means and maxima of voxel kinetic estimates, averaged over replicates, are shown as ${\bar{\mu }}_{•}$ and ${\bar{\mu }}_{{}^{{}_{\vee }}}$. Mean of voxel standard deviations, averaged over replicates $\left({\bar{\sigma }}_{•}\right)$ its standard error $\left(\overset{\bar{}}{SE}{\;}_{•}\right)$ and the overall RMSE values, see (20), are reported as a percentage of the maximum ${\bar{\mu }}_{{}^{{}_{\vee }}}$. Estimates ($\hat{a}$, $\hat{b}$, $\hat{c}$) correspond to (19).

Parameters	Brain FDG						Breast FLT					
	Vb	Vd	Kd	Ki	MTT	Ki/K1	Vb	Vd	Kd	Ki	MTT	Ki/K1
Mean $\left({\bar{\mu }}_{•}\right)$	0.04	0.50	0.23	0.01	4.19	0.02	0.22	0.23	0.04	0.01	4.58	0.12
Max $\left({\bar{\mu }}_{{}_{\vee }}\right)$	0.12	1.11	0.56	0.02	8.71	0.17	1.96	1.54	0.19	0.07	8.36	0.59
${\bar{\sigma }}_{•}$	20.00	11.17	14.59	5.47	10.41	7.22	2.61	3.81	11.14	1.52	22.73	12.52
$\overset{\bar{}}{SE}{\;}_{•}$	1.50	0.73	1.12	0.33	0.57	0.71	0.19	0.26	0.87	0.10	1.38	1.00
$\hat{a}$	1.11	1.08	1.08	1.16	1.14	1.12	1.04	1.08	1.12	1.07	1.20	1.18
$\hat{b}$	0.95	0.95	0.92	1.10	1.04	1.00	0.90	0.98	1.00	0.98	1.14	1.08
$\hat{c}$	0.97	0.97	0.94	1.12	1.06	1.04	0.91	0.99	1.03	0.99	1.16	1.11
${\text{RMSE}}_{•}^{y}$	6.17	2.38	4.33	1.50	2.89	3.69	0.69	1.00	4.14	0.35	8.43	5.13
${\text{RMSE}}_{•}^{z}$	7.10	2.95	5.29	1.41	2.79	4.04	0.91	1.11	4.35	0.40	8.04	5.09
${\text{RMSE}}_{•}^{z_{*}}$	8.31	3.65	6.13	1.76	3.41	4.55	1.08	1.35	5.08	0.50	9.40	5.88

**Table 2:** Reliability of Bootstrap Estimates of Standard Deviations for ROI means of mapped Kinetics. Note approximately 700 ROIs are considered in each case, see Fig 4. Means and maxima of ROI-averaged kinetic parameters, averaged over replicates, are shown as ${\bar{\mu }}_{•}$ and ${\bar{\mu }}_{{}_{\vee }}$. Mean ROI standard deviation, averaged over replicates $\left({\bar{\sigma }}_{•}\right)$, its standard error $\left(\overset{\bar{\;}}{SE}{\;}_{•}\right)$ and the overall RMSE values, computed by ROI version of (20), are reported as a percentage of the maximum $\left({\bar{\mu }}_{{}_{\vee }}\right)$. Estimates ($\hat{a}$, $\hat{b}$, $\hat{c}$) are based on the ROI version of (19).

Parameters	Brain FDG						Breast FLT					
	Vb	Vd	Kd	Ki	MTT	Ki/K1	Vb	Vd	Kd	Ki	MTT	Ki/K1
Mean $\left({\bar{\mu }}_{•}\right)$	0.04	0.60	0.26	0.01	4.56	0.04	0.27	0.26	0.04	0.01	4.24	0.14
Max $\left({\bar{\mu }}_{{}_{\vee }}\right)$	0.09	1.08	0.49	0.02	8.58	0.18	1.63	1.30	0.17	0.07	8.04	0.52
${\bar{\sigma }}_{•}$	19.93	8.97	12.04	4.21	6.47	5.54	1.82	2.54	7.00	0.85	11.58	7.24
$\overset{\bar{}}{SE}{\;}_{•}$	1.48	0.61	0.91	0.27	0.40	0.46	0.12	0.17	0.50	0.06	0.76	0.52
$\hat{a}$	1.18	1.14	1.14	1.22	1.22	1.18	1.27	1.18	1.23	1.10	1.24	1.23
$\hat{b}$	1.12	1.08	1.08	1.21	1.18	1.14	1.04	1.08	1.11	1.07	1.20	1.16
$\hat{c}$	0.73	0.82	0.70	0.77	0.98	0.53	0.71	0.63	0.58	0.57	0.87	0.55
${\text{RMSE}}_{•}^{y}$	5.55	1.80	3.25	1.12	1.62	2.25	0.38	0.54	1.92	0.17	3.36	2.27
${\text{RMSE}}_{•}^{z}$	5.99	2.08	3.47	1.16	1.77	2.32	0.54	0.64	2.23	0.19	3.51	2.44
${\text{RMSE}}_{•}^{z_{*}}$	12.39	4.17	8.21	2.36	3.13	7.91	1.22	2.04	6.83	0.85	7.35	8.08

**Table 3:** Reliability of Bootstrap Estimates of voxel Standard Deviations for mapping Kinetics in 1-D based on FBP and ML reconstructed data. Means and maxima of voxel kinetic estimates, averaged over replicates, are shown as ${\bar{\mu }}_{•}$ and ${\bar{\mu }}_{{}_{\vee }}$. Mean of voxel standard deviations, averaged over replicates $\left({\bar{\sigma }}_{•}\right)$, its standard error $\left(\overset{\bar{\;}}{SE}{\;}_{•}\right)$ and the overall RMSE values, see (20), are reported as a percentage of the maximum $\left({\bar{\mu }}_{{}_{\vee }}\right)$. Estimates ($\hat{a}$, $\hat{b}$, $\hat{c}$) correspond to (19).

Tracer	Parameters	FBP						ML					
		Vb	Vd	Kd	Ki	MTT	Ki/K1	Vb	Vd	Kd	Ki	MTT	Ki/K1
FDG	Mean $\left({\bar{\mu }}_{•}\right)$	0.05	0.49	0.26	0.01	3.03	0.02	0.05	0.49	0.26	0.01	3.06	0.02
	Max $\left({\bar{\mu }}_{{}_{\vee }}\right)$	0.26	0.95	1.07	0.02	4.36	0.07	0.26	0.95	1.07	0.02	4.36	0.07
	${\bar{\sigma }}_{•}$	4.68	7.11	4.38	2.57	14.16	7.56	3.68	6.79	3.63	2.39	12.04	6.66
	$\overset{\bar{}}{SE}{\;}_{•}$	0.20	0.22	0.18	0.09	0.41	0.28	0.15	0.21	0.14	0.09	0.37	0.22
	$\hat{a}$	1.06	1.16	1.07	1.03	1.19	1.12	1.09	1.17	1.11	1.04	1.18	1.15
	$\hat{b}$	1.12	1.20	1.13	1.04	1.22	1.16	1.06	1.20	1.10	1.04	1.22	1.15
	$\hat{c}$	1.16	1.23	1.16	1.07	1.25	1.19	1.09	1.23	1.13	1.07	1.26	1.18
	${\text{RMSE}}_{•}^{y}$	29.7	31.4	26.9	17.9	31.3	29.2	28.0	32.5	25.9	17.2	29.1	26.7
	${\text{RMSE}}_{•}^{z}$	33.1	34.3	30.6	23.7	33.7	32.9	34.2	35.9	30.9	24.7	34.6	31.4
	${\text{RMSE}}_{•}^{z_{*}}$	41.9	42.3	39.5	34.2	42.2	42.3	44.3	44.5	40.5	36.0	42.9	41.6
FLT	Mean $\left({\bar{\mu }}_{•}\right)$	0.18	0.71	0.16	0.02	3.46	0.12	0.18	0.71	0.16	0.02	3.47	0.12
	Max $\left({\bar{\mu }}_{{}_{\vee }}\right)$	1.04	2.17	0.36	0.05	7.50	0.61	1.04	2.17	0.36	0.05	7.57	0.61
	${\bar{\sigma }}_{•}$	2.01	1.93	4.26	1.72	9.69	2.93	1.62	1.83	3.65	1.66	9.23	2.45
	$\overset{\bar{}}{SE}{\;}_{•}$	0.08	0.08	0.17	0.08	0.46	0.15	0.06	0.08	0.15	0.07	0.44	0.11
	$\hat{a}$	1.02	0.98	1.01	0.99	1.11	1.08	1.05	1.01	1.06	1.03	1.13	1.14
	$\hat{b}$	1.20	0.98	1.05	0.98	1.12	1.11	1.22	0.97	1.03	0.99	1.13	1.13
	$\hat{c}$	1.23	1.01	1.08	1.02	1.18	1.17	1.25	1.01	1.06	1.03	1.20	1.18
	${\text{RMSE}}_{•}^{y}$	25.4	31.6	27.2	37.8	44.4	56.0	22.2	30.6	24.9	33.7	44.2	50.2
	${\text{RMSE}}_{•}^{z}$	33.3	34.7	29.3	41.3	46.0	59.0	34.6	35.0	28.8	39.2	47.5	58.0
	${\text{RMSE}}_{•}^{z_{*}}$	41.3	45.6	38.7	51.2	57.6	72.7	42.6	44.8	38.4	48.8	59.3	71.4

**Table 4:** Analysis of Voxel-level Error Characteristics across all Simulations. The coefficients are for the models in (22) and (23). The *R*^2^-values only reported for (23). Corresponding values for (22), are uniformly high (in excess of 90%).

		FDG						FLT					
		Vb	Vd	Kd	Ki	MTT	Ki/K1	Vb	Vd	Kd	Ki	MTT	Ki/K1
FBP (2D)	$\hat{\alpha }$	0.86	0.88	0.86	0.94	0.92	0.90	0.86	0.91	0.90	0.92	0.95	0.92
	$\hat{\beta }$	0.88	0.89	0.87	0.95	0.93	0.93	0.88	0.93	0.92	0.93	0.97	0.94
	${\hat{\gamma }}_{1}$	0.58	0.63	0.65	0.59	0.55	0.48	0.79	0.63	0.59	0.63	0.90	0.75
	*R* ^2^	0.93	0.94	0.94	0.90	0.33	0.61	0.97	0.94	0.85	0.94	0.97	0.91
FBP (1D)	$\hat{\alpha }$	1.06	1.03	1.05	1.01	1.03	1.03	1.18	1.00	1.03	0.99	1.01	1.03
	$\hat{\beta }$	1.10	1.06	1.09	1.04	1.06	1.07	1.21	1.04	1.07	1.03	1.06	1.08
	${\hat{\gamma }}_{1}$	0.73	0.65	0.70	0.85	0.65	0.47	0.77	0.92	0.86	0.85	1.11	0.56
	*R* ^2^	0.91	0.69	0.91	0.98	0.15	0.72	0.97	0.91	0.94	0.89	0.83	0.37
ML (1D)	$\hat{\alpha }$	0.97	1.02	0.99	1.00	1.03	1.00	1.16	0.96	0.97	0.96	1.00	0.99
	$\hat{\beta }$	1.01	1.05	1.02	1.03	1.07	1.03	1.19	1.00	1.00	1.00	1.05	1.04
	${\hat{\gamma }}_{1}$	0.64	0.67	0.57	0.83	0.07	0.43	0.65	0.92	0.83	0.85	1.11	0.55
	*R* ^2^	0.89	0.55	0.83	0.98	0.00	0.73	0.90	0.92	0.92	0.88	0.81	0.40

## Footnotes

1 The logarithmic transform is used for variance stabilization in fitting.
